# An immunoinformatic approach driven by experimental proteomics: *in silico* design of a subunit candidate vaccine targeting secretory proteins of *Leishmania donovani* amastigotes

**DOI:** 10.1186/s13071-020-04064-8

**Published:** 2020-04-15

**Authors:** Md Anik Ashfaq Khan, Jenifar Quaiyum Ami, Khaledul Faisal, Rajashree Chowdhury, Prakash Ghosh, Faria Hossain, Ahmed Abd El Wahed, Dinesh Mondal

**Affiliations:** 1 Nutrition and Clinical Services Division, International Centre for Diarrheal Disease Research, Bangladesh, Dhaka, 1212 Bangladesh; 2 Infectious Diseases Division, International Centre for Diarrheal Disease Research, Bangladesh, Dhaka, 1212 Bangladesh; 3 Microbiology and Animal Hygiene Division, Georg-August-University Goettingen, Burckhardtweg 2, 37077 Göttingen, Germany

**Keywords:** Visceral leishmaniasis, *In silico* vaccine design, Reverse vaccinology using proteomics

## Abstract

**Background:**

Visceral leishmaniasis (VL) caused by dimorphic *Leishmania* species is a parasitic disease with high socioeconomic burden in endemic areas worldwide. Sustaining control of VL in terms of proper and prevailing immunity development is a global necessity amid unavailability of a prophylactic vaccine. Screening of experimental proteome of the human disease propagating form of *Leishmania donovani* (amastigote) can be more pragmatic for *in silico* mining of novel vaccine candidates.

**Methods:**

By using an immunoinformatic approach, CD4+ and CD8+ T cell-specific epitopes from experimentally reported *L. donovani* proteins having secretory potential and increased abundance in amastigotes were screened. A chimera linked with a Toll-like receptor 4 (TLR4) peptide adjuvant was constructed and evaluated for physicochemical characteristics, binding interaction with TLR4 in simulated physiological condition and the trend of immune response following hypothetical immunization.

**Results:**

Selected epitopes from physiologically important *L. donovani* proteins were found mostly conserved in *L. infantum*, covering theoretically more than 98% of the global population. The multi-epitope chimeric vaccine was predicted as stable, antigenic and non-allergenic. Structural analysis of vaccine-TLR4 receptor docked complex and its molecular dynamics simulation suggest sufficiently stable binding interface along with prospect of non-canonical receptor activation. Simulation dynamics of immune response following hypothetical immunization indicate active and memory B as well as CD4+ T cell generation potential, and likely chance of a more Th1 polarized response.

**Conclusions:**

The methodological approach and results from this study could facilitate more informed screening and selection of candidate antigenic proteins for entry into vaccine production pipeline in future to control human VL.
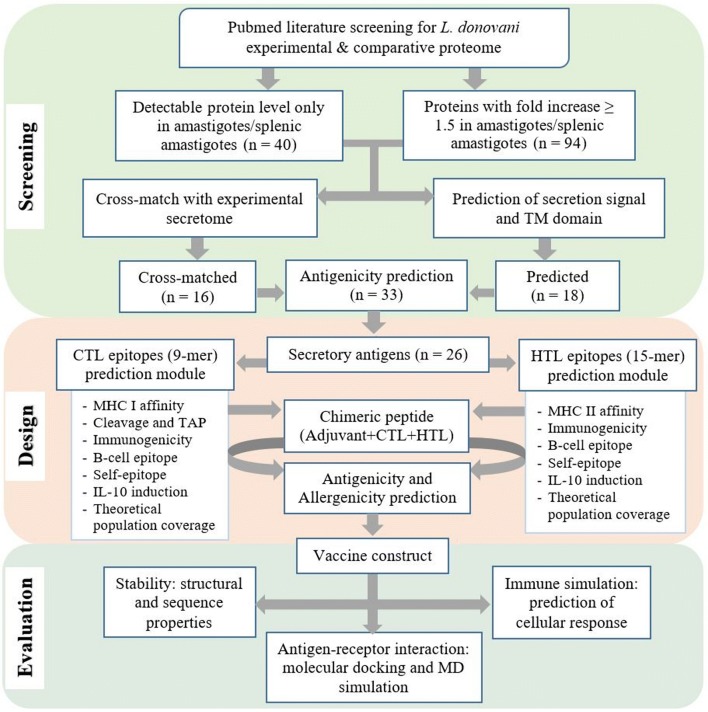

## Background

*Leishmania* spp. are obligate intracellular pathogens of phagocytic host cells. Two species, *Leishmania donovani* and *L. infantum* cause visceral leishmaniasis (VL), a neglected tropical disease and second only to malaria in parasitic cause of death. With a chance of case fatality of 100% in an inadequate treatment scenario, over 90% of VL cases occur in relatively poor communities of Bangladesh, India, Sudan, South Sudan, Ethiopia and Brazil [1]. The VL elimination program known as Kala-azar Elimination Programme (KEP) has contributed to a remarkable decline in the incidence of VL over recent years in the Indian subcontinent and now it is approaching the maintenance phase of VL elimination [2]. However, sustained elimination cannot be possible without proper and prevailing immunity development in the endemic population against *Leishmania* parasites in the post-elimination era due to the chance of reservoir mediated re-emergence of the disease [3]. A vaccination strategy can induce long-term protection with proper immunity in order to prevent development of disease in the most economical way, regardless of its mode of implementation.

In recent years, enormous progress has been made in the design of vaccines against leishmaniasis using live-attenuated or killed parasites, cellular extracts, and individual and/or recombinant antigens of parasites. The first-generation vaccine, which includes live-attenuated, killed and fractionated parasites, is the only class of human prophylactic VL vaccine that entered phase III clinical trials so far. However, this vaccine failed to achieve satisfactory results [4]. The second-generation vaccines are produced from recombinant *Leishmania* antigens (single peptides/polypeptides). Among several approaches, LEISH-F3, a multicomponent vaccine formulated with GLA-SE adjuvant showed promising results in phase I as a robust immune response inducer in healthy people [5]. Earlier, LEISH-F1 in combination with MPL-SE adjuvant also showed strong antigen-specific immune response in healthy people living in a *L. donovani* endemic area [6]. More recently, a third-generation DNA vaccine approach that employed simian adenovirus expressing a novel synthetic gene encoding *Leishmania* antigens, hence termed as ChAd63-KH, has shown potentiality to be a safe and immunogenic therapeutic vaccine for human VL and post kala-azar dermal leishmaniasis (PKDL) in a phase I trial [7]. Despite the ongoing progresses in vaccine development, the priority objective has not yet been achieved, i.e. the development of safe, effective, durable and low-cost prophylactic vaccine for human visceral leishmaniasis [8].

Besides producing memory lymphocytes towards a long-term immunity pathway, an ideal vaccine against *Leishmania* will stimulate parasite-specific cellular immunity that include a strong Th1 response to eliminate infections. In this regard, the use of epitopes or epitope-containing peptides is advantageous since epitopes can be evaluated for immuno-recognition and epitope-specific response. Since epitopes/peptides themselves remain poorly immunogenic, the approaches that have been gaining interest are based on the development of peptide-based formulations in combination with potent adjuvant components (peptide, lipids, virus particles, nanoparticles etc.) [9]. However, mapping of epitopes in immunogenic proteins remains crucial in peptide vaccine development. In addition to *in vitro* methods of epitope mapping such as phage display library, immunodominance and peptide competition assays, immunoinformatic mapping can be a powerful approach to facilitate screening of desired epitopes in immunogenic proteins [9]. Recent findings of leishmaniasis vaccine research also suggest that *in silico* predicted MHC class I and class II restricted epitope-containing peptides derived from *Leishmania* antigens alone, as a cocktail, as a chimeric peptide or in combination with adjuvant can be substantially immunogenic *in vitro* and/or *in vivo* [10–13]. Thus, the application of immunoinformatics-based pipeline can facilitate large-scale screening of peptide epitopes from *Leishmania* proteome for rational design of potent vaccines.

While derivation of potentially immunogenic peptides can be performed by analyzing (*in vitro* and/or *in silico*) either the whole parasite proteome, proteins known to elicit immunological outcome, or the known peptide libraries [9], two essential criteria have been suggested for consideration to initially select potential vaccine antigens for leishmaniasis: (i) known antigen that is expressed in the disease-causing mammalian stage of the parasite; and (ii) selected adjuvants that elicit a cellular, Th1-biased immune response for the immunizations in humans [14]. The human stage-associated *Leishmania* proteins that facilitate intracellular survival and infective process of the parasite thus constitute attractive targets for anti-*Leishmania* vaccine design. In order to adapt in mammalian host, the promastigote stage of *Leishmania* undergoes morphological and metabolic changes when transformed into amastigote stage upon entry and invasion. This is accompanied by a cascade of programmed changes in mRNA abundance, translation rate, and/or protein processing. However, interpretation of *Leishmania* transcriptome is likely controversial on whether relative changes in mRNA abundance is substantial [15, 16], constitutive or negligible [17–19]. Moreover, stage-specific upregulation for some transcripts [20] does not necessarily reflect in altered functional protein profile because of post-transcriptional [21] and post-translational regulation [22] evident for *Leishmania* species. While mRNA abundance may not be a perfect indicator of protein levels in eukaryotes [19, 23, 24], the relationship between mRNA and protein abundance is suggested to be dynamically changing as *L. donovani* adapts to amastigote condition, with correlation in changes for only a small proportion [22]. Moreover, the changes in protein level can also vary between clinical and cultured amastigotes [25]. In this scenario, experimentally evaluated proteome analysis can better highlight the key changes, which have important implications for diagnostics, drug target identification and vaccine design.

Reverse vaccinology [26] has been becoming increasingly popular in supported vaccine design by the combined use of genomics, transcriptomics, proteomics and immunoinformatics. Here, we propose an approach to design a subunit vaccine based exclusively on mass spectrometry (MS)-driven comparative proteomic information associated with amastigotes, since genome/transcriptome information can be deviant as *Leishmania* adapts to amastigote condition. Invasion and survival of *L. donovani* in the mammalian host largely involves the export of virulence factors and immune-modulatory components into the host cytosol. However, constant exposure of these secretory proteins to host immune system can lead to immunological tolerance and strong parasite-specific humoral response, which can be problematic for vaccine design. Hence, we limited our focus on rational screening of immunogenic T cell-specific epitopes in such secretory proteins, which have substantial coverage of endemic population as well. We then combined the epitopes into a single recombinant protein molecule. We explored into the physicochemical properties and receptor binding interaction of the multi-epitope vaccine, followed by molecular dynamics simulation of the vaccine-receptor complex and simulation of immune response. Overall, we showed that the experimental proteome data-driven immunoinformatic approach can facilitate informed screening of potential subunit vaccine candidates from truly produced human stage-associated parasitic proteins of pathological/physiological importance. The immunogenic potential evaluated *in silico* can also provide rationality for experimental validation of the modeled subunit vaccine.

## Methods

**Fig. 1:**
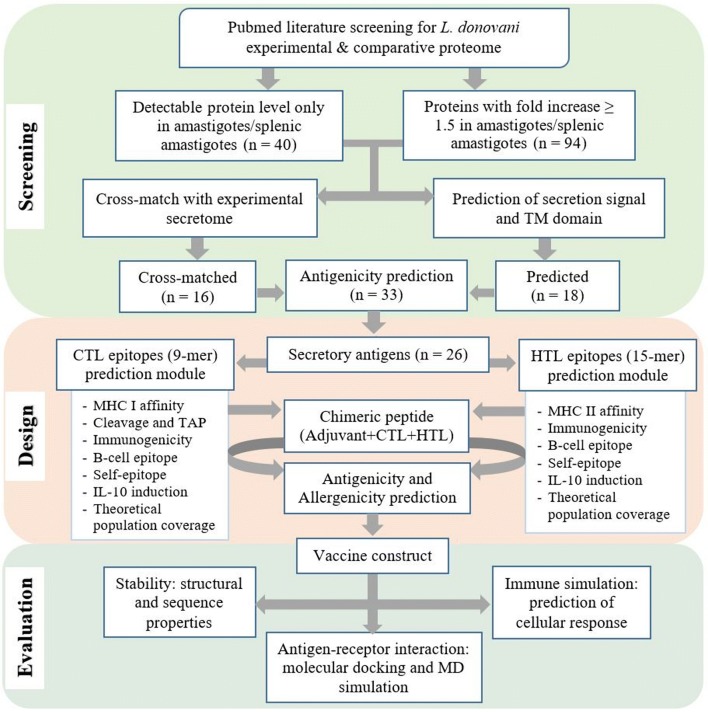
Methodological flowchart in multi-epitope subunit vaccine design

All the computational tasks using online and offline tools in this study were carried out with the concurrent version of the tools between September 2018 and December 2018. Graphpad Prism v.7 software was used for descriptive data calculation, comparison of means and to reproduce graphs using software generated numeric values when applicable. A two-tailed *P*-value of < 0.05 was considered as significant. The methodological flowchart of the study is given in Fig. 1. The details of the methodological steps are given in Additional file 1: Text S1.

### Antigen selection

Literature reports on the proteome profile of *L. donovani* were screened in the PubMed (NCBI) database to index articles published between year 2000 and 2018, and reports on MS-driven comparative abundance of proteins in promastigotes and amastigotes were selected to generate a local database of proteins. Sequences of identical or closely similar *L. donovani* proteins, which had more abundance in amastigotes, were retrieved. An MS-derived secretome profile of *L. donovani* was also retrieved from the published literature [27]. Secretory proteins in the local database were screened by both cross-matching the secretome and proteome data using ViroBLAST [28], as well as by evaluating combined prediction of SignalP [29], SecretomeP [30] and TMHMM [31]. Antigenicity of the potential secretory proteins was estimated using ANTIGENpro [32] and VaxiJen [33] programs.

### Epitope screening

For screening of cytotoxic T-lymphocyte (CTL, 9-mer) epitopes and helper T-lymphocyte (HTL, 15-mer) epitopes, both affinity and allele coverage were considered. Initially, CTL and HTL epitopes were screened for above cut-off prediction scores in NetCTL [34] and lower percentile rank in IEDB (Immune Epitope Database) recommended MHC-II consensus module (http://tools.iedb.org/tcell/), respectively. Epitopes that are superior in human leukocyte antigen (HLA) cross-allele coverage were preliminarily selected. Then, both CTL and HTL epitope sets were filtered through specific (MHC-I and MHC-II modules of IEDB) and common (VaxiJen) immunogenicity prediction tools. CTL epitopes were further filtered in TAPpred [35] for more accurate prediction of TAP transporter binder. As per requirement of a proper anti-*Leishmania* immune response, T cell epitopes containing B cell recognition region (predicted by Bepipred [36]) were excluded, and all IL-10 inducing epitopes (predicted by IL-10Pred [37]) were removed. BLAST search against the non-redundant human protein database was carried out to rule out self-epitopes, while search against the RefSeq [38] protein database of *Leishmania* was performed to find out the conservancy of the epitopes in other *Leishmania* species.

In order to calculate the theoretical population coverage (TPC) (http://tools.iedb.org/population/) of each epitope, binding affinity to HLA allele-specific MHC molecules was set at percentile rank cut-off of 1.0 for CTL epitopes (IEDB recommended), and IC_50_ cut-off of 100 nM for HTL epitopes (10-times lower than the IEDB recommended value). Our target was to reach more than 90% population coverage by both CTL and HTL epitope sets in each of three most endemic areas of VL (India, Sudan and Brazil) with maximum number of alleles per epitope, while keeping the subunit length at minimum. In accordance, CTL epitopes with minimum TPC of 40% and at least eight HLA alleles were selected for vaccine construction. On the other hand, most of the screened HTL epitopes had more than 80% coverage in our observation, and therefore HTL epitopes having greater than 90% coverage were selected.

### Chimeric vaccine construction and evaluation

The vaccine construct was arranged by joining the CTL and HTL epitopes with linkers [39, 40], and preceded by a synthetic peptide adjuvant [41]. The selection of chimeric arrangement was based on antigenicity (ANTIGENpro and VaxiJen) and allergenicity (AlgPred [42] and AllerTOP [43]) scores, retaining of cleavage, TAP transporter- and MHC-binding propensity of target epitopes, and generation of none/least number of non-specific and/or IL-10 inducing epitopes due to recombination. The capability to induce IFN-γ and IL-10 by the chimera was predicted by scanning in IFNepitope [44] and IL-10Pred module, respectively. Simulation of immune response based exclusively on the chimeric construct was performed in C-ImmSim [45] server, whereas two previously reported candidate *Leishmania* vaccine peptides [46, 47] were used to evaluate whether C-ImmSim prediction corroborates to the dynamicity of antigenic constructs. For structural analysis, the tertiary structure of the construct was produced in I-TASSER [48] modeling server followed by refinement using YASARA [49] force-field and GalaxyRefine [50] web tools. Stability of vaccine construct, a prerequisite for antigen processing, was assessed using physicochemical features predicted by both sequence-based (ProtParam [51]) and structural (SCooP [52] and CamSol [53]) analysis tools. Furthermore, chimeric vaccine-specific linear (Bepipred and BCPREDS [54]) and conformational (Ellipro [55]) B cell epitopes were predicted. The structural model was used to dock to Toll-like receptor 4 by using ClusPro [38] docking server and the binding interactions were analyzed. Molecular dynamics (MD) simulation was performed by using Desmond v5.3 (Schrödinger, LLC, New York, USA) software to check the conformational stability of vaccine-receptor docked complex.

### *In silico* cloning

To validate the immunoinformatic findings, evaluation of immuno-reactivity through serological analysis is the preliminary step and this requires expression of the candidate vaccine. For this purpose, codon optimization was carried out by using JCAT [56]. A cloning model was then developed for this construct by using SnapGene (GSL Biotech, California, USA) tool and by inserting the optimized coding sequence into a plasmid vector.

## Results

### Screening of secretory amastigote proteins from experimental studies

Following literature screening, three out of 28 PubMed indexed experimental studies on *L. donovani* proteome were considered for the study. Two of the studies compared proteomic abundance of promastigotes with that of amastigotes as *Leishmania* adapts to the changes in conditions resembling the host [57, 58], while the other one compared splenic amastigotes to axenic amastigotes [25]. A total of 118 out of 134 proteins, which had a relative increase of at least 1.5-fold or were reported exclusively in the protein profile of amastigotes and/or splenic amastigotes, were found to have an identity of 90% or above for absolute query coverage with *L. donovani* proteins of similar functional annotations. After cross-matching of this group of proteins to 151 *L. donovani* secretory proteins revealed experimentally [27], 16 proteins were found to have an identity percentage and query coverage of 96.75 ± 1.1% and 99.44 ± 1.13%, respectively. Based on the presence of classical or non-classical secretion signal sequences along with minimum (no more than one) transmembrane helices, one common and an additional 17 secretory proteins were included to the pool. Among 33 amastigote-associated potential secretory proteins, 26 were selected based on their antigenicity probability scores of ≥ 0.5 as predicted by both ANTIGENpro and VaxiJen (Table 1, Additional file 2: Data S1).

**Table 1:** List of 26 amastigote proteins, their secretory potential, antigenicity scores and relevant reports

SN.	UniProt ID	Name	Secretion annotation	Status in amastigote	Antigenicity (VaxiJen/ ANTIGENpro)	Reference	Association of target protein or its homolog with parasite and/or infection	Report on immune activity against similar protein of *Leishmania* (source spp.)
1	E9BT80	Elongation factor 2	Experimental	Increased	0.517/0.614	[25]	Protein synthesis; associated with increased drug resistance [95]	Th1 pathway stimulatory *in vitro* and protective *in vivo* (*L. donovani*) [96]
2	E9BTS3	Chaperonin HSP60, mitochondrial	Experimental	Specific^a^	0.568/0.701	[57]	Protein folding/re-folding; upregulated in axenic amastigotes [97, 98]	Immunoreactive in sera of human (*L. major*) and dog (*L. infantum*) [99, 100]
3	A4GVE9	Eukaryotic translation initiation factor 5A	Experimental	Specific^a^	0.575/0.868	[57]	Viability and proliferation [101]	Cross-protective *in vivo* (*L. braziliensis*) [102, 103]
4	O43941	Protein phosphatase-2C	Experimental	Specific^a^	0.584/0.910	[57]	Potential to regulate stress signal; upregulated in drug resistance phenotype [104]	Pro-inflammatory *in vitro* (*L. donovani*) [105]
5	E9BT68	Stress-inducible protein STI1 homolog	Experimental	Increased	0.634/0.908	[25]	Co-chaperone; preferential expression in macrophages [106]	Unknown
6	E9BK18	Heat-shock protein hsp70, putative	Experimental	Increased	0.521/0.916	[25]	Protein folding; drug resistance; phosphoprotein activity [107]	Immunoreactive in sera of human (*L. major*) and dog (*L. infantum*) [108, 109]
7	E9BIV4	Proteasome endopeptidase complex	Experimental	Increased	0.623/0.676	[58]	Growth and intra-cellular survival; regulation of microbicidal activity [110]	Immuneproteomic detection (*L. infantum*) [111]
8	P17804	Heat-shock 70 kDa protein	Experimental	Specific^a^	0.534/0.920	[57]	protein folding; upregulated in drug resistance phenotype; Phosphoprotein activity [107]	Immunoreactive in sera of human (*L. major*) and dog (*L. infantum*) [108, 109]
9	B5APK3	Nucleoside diphosphate kinase	Experimental	Specific^a^	0.691/0.762	[57]	Metabolism (purine salvage pathway); preservation of host-cell integrity [112]	Immunoreactive *in vitro* (*L. amazonesis*) [112]
10	E9BTS2	Chaperonin HSP60, mitochondrial	Experimental	Increased	0.956/0.791	[25]	Protein folding/re-folding; increased expression in axenic amastigotes [97, 98]	Immunoreactive in sera of human (*L. major*) and dog (*L. infantum*) [99, 100]
11	E9BI90	Glutathione peroxidase	Experimental	Increased	0.564/0.886	[25]	ROS detoxification (identical to type II tryparedoxin peroxidase) [113]	Unknown
12	E9BDB8	Uncharacterized protein (containing META domain)	Predictive (non-classical)	Increased	0.503/0.872	[25]	Possible protection from intracellular stress (predicted from sequence homology to Q8MTW1) [114]	Unknown
13	E9BI76	Protein disulfide isomerase, putative	Predictive (classical)	Increased	0.513/0.500	[25]	Potential role in growth and virulence; high expression in amastigotes [115]	Th1 pathway stimulatory *in vitro* and cellular immunity inducive *in vivo* (*L. donovani*) [116]
14	E9BKN2	Cysteine peptidase C (CPC)	Predictive (classical)	Increased	0.518/0.948	[25]	Virulence in host via regualtion of parasite secreted proteins [117]	Protective response and antigenicity *in vivo* by recombinatorial vaccine (*L. infantum*) [118]
15	E9BJQ0	Uncharacterized protein	Predictive (non-classical)	Specific^a^	0.520/0.739	[57]	Unknown function	Unknown
16	E9BS02	Thioredoxin-like protein	Predictive (non-classical)	Increased	0.536/0.749	[25]	Antioxidant activity (general function)	Unknown
17	E9BQ40	Uncharacterized protein (containing alpha/beta hydrolase domain)	Predictive (non-classical)	Increased	0.557/0.740	[25]	Unknown function; upregulation of similar domain containing protein in amastigotes [119]	Unknown
18	E9BUW4	Mkiaa0324 protein-like protein (serine/arginine repetitive matrix protein 2)	Predictive (non-classical)	Increased	0.590/0.890	[25]	Unknown function; upregulated in drug resistance phenotype [120]	Unknown
19	E9BBJ4	Uncharacterized protein (containing Complex1_LYR_1 motif)	Predictive (non-classical)	Specific^a^	0.597/0.632	[57]	Possible role in metabolic switching by regulating glucose uptake (predicted) [121]	Unknown
20	E9BNJ3	Uncharacterized protein (containing RNA recognition motif RRM_8)	Predictive (non-classical)	Increased	0.600/0.934	[58]	Unknown function	Unknown
21	P23223	Leishmanolysin	Predictive (non-classical)	Increased	0.609/0.535	[25]	Host invasion, phagocytosis and immune-evasion [122]	Th1 dominant and protective response *in vivo* (*L. donovani*) [123]
22	E9B882	Fructose-1,6-bisphosphatase, cytosolic, putative	Predictive (non-classical)	Increased	0.639/0.657	[25]	Virulence and replication inside host [124]	Unknown
23	E9B833	Ubiquitin-conjugating enzyme e2, putative	Predictive (non-classical)	Increased	0.649/0.825	[25]	Ubiquitin conjugation system; proteasomal degradation of proteins [125]	Unknown
24	Q95WR6	Cysteine protease	Predictive (classical)	Increased	0.658/0.792	[58]	Parasite growth and host pathogenesis; upregulated in drug resistance phenotype [126]	Immunoreactive in plasma of human (*L. donovani*) [127]
25	E9BKM5	Lipophosphoglycan biosynthetic protein, putative	Predictive (classical)	Increased	0.668/0.733	[25]	Processing and transport of secreted proteins; chaperone; endoplasmin homolog; upregulated in amastigotes; heparin binding [128, 129]	Immunogenic *in vivo* and immunoreactive in sera of human (*L. major*) [130]
26	E9BED5	Cysteine peptidase A (CPA)	Predictive (classical)	Increased	0.670/0.939	[25]	Host-parasite interaction [131]	Th1 pathway stimulatory and cross-protective *in vivo* (*L. infantum*) [10]

^a^Found to be present at detectable level

### Multi-epitope subunit *L. donovani* vaccine: construction and properties

A total of 79 CTL 9-mer epitopes were initially screened in NetCTL. Among them, only nine epitopes from six proteins were predicted to be non-self, highly immunogenic and high-to-moderate TAP-transporter binder non-B cell epitopes. These epitopes covered theoretically, an average of 66.46 ± 7.88% and a cumulative of 98.57% of the world population. Similarly, HTL 15-mer epitopes were screened to ensure both affinity and coverage. Fourteen selected HTL epitopes from eight proteins were finally predicted to be non-self, highly immunogenic non-B cell epitopes, with a mean theoretical coverage of 96.62 ± 1.35% and a cumulative of 99.52% of the world population. All the CTL and HTL epitopes except for H2-10 and H2-13 were conserved (100% identical) in *L. infantum*, whereas, less conservancy was found in representative proteins of *L. major* (13/23) and *L. mexicana* (10/23). Properties of individual CTL and HTL epitopes are given in Tables 2 and 3, respectively.

**Table 2:** List of MHC I epitopes with characteristic affinity and coverage

Epitope No.	Epitope sequence	Protein ID	Starting position	HLA supertype coverage	Tappred binding	VaxiJen score	IEDB class I score	No. of HLA alleles	World TPC (%)	Epitope conservancy^a^ in other *Leishmania* spp. in RefSeq database	Self^b^
H1-01	LLYGGIFCY	E9B882	260	A1, A2, A3, A26, B58, B62	High	2.40	0.26	8	41.37	*L. major*, *L. infantum*, *L. braziliensis*, *L. mexicana*, *L. guyanensis*	No
H1-02	KIYANWPTY	E9BS02	189	A3, A26, B58, B62	High	1.41	0.25	15	63.11	*L. major*, *L. infantum*, *L. mexicana*	No
H1-03	FVAYFRTPL	E9BS02	77	A2, B7, B8, B39, B62	Medium	0.91	0.19	21	85.48	*L. major*, *L. infantum*, *L. mexicana*	No
H1-04	FVKWNFTAF	E9BI90	122	A24, A26, B7, B8, B62	Medium	1.08	0.31	16	67.98	*L. infantum*	No
H1-05	FMHVYTTHF	E9BIV4	119	A1, B8, B58, B62	Medium	0.77	0.14	19	83.44	*L. major*, *L. infantum*, *L. mexicana*	No
H1-06	HVYTTHFAY	E9BIV4	121	A1, A3, A26, B8, B58, B62	High	0.73	0.23	19	73.51	*L. major*, *L. infantum*, *L. mexicana*	No
H1-07	YVAFVERLY	E9BQ40	345	A1, A3, A26, B62	Medium	1.39	0.30	13	51.83	*L. major*, *L. infantum*, *L. mexicana*, *L. braziliensis*, *L. panamensis*	No
H1-08	RVAAALRIY	E9BUW4	58	A1, A3, A26, B58, B62	High	0.77	0.20	12	51.08	*L. major*, *L. infantum*, *L. mexicana*	No
H1-09	ATYAALLPL	E9BUW4	450	A2, B7, B58, B62	High	0.77	0.05	19	80.33	*L. major*, *L. infantum*, *L. mexicana*, *L. braziliensis*, *L. panamensis*	No

^a^100% identity for absolute query cover

^b^identity threshold in human for amino acids > 7 (78%)

**Table 3:** List of MHC II epitopes with characteristic affinity and coverage

Epitope no.	Selected HLA epitopes	Protein ID	Starting position	Vaxijen score	IEDB class II rank	IL-10	World TPC (%)	Epitope conservancy^a^ in other *Leishmania* spp. in RefSeq database	Self^b^
H2-01	QDCKFVLVKAAAPAA	E9BDB8	325	0.77	6.12	No	98.71	*L. major*, *L. infantum*	No
H2-02	AAYYIKAAERIAAKG	E9BI76	321	0.94	4.67	No	97.15	*L. major*, *L. infantum*	No
H2-03	TFVKWNFTAFLVDKD	E9BI90	121	1.19	8.91	No	97.76	*L. infantum*	No
H2-04	LGTTFVKWNFTAFLV	E9BI90	118	0.97	9.34	No	97.46	*L. infantum*	No
H2-05	TTFVKWNFTAFLVDK	E9BI90	120	1.06	8.31	No	97.76	*L. infantum*	No
H2-06	LTKLFRYKSSRSESE	E9BKM5	486	0.82	6.18	No	95.45	*L. major*, *L. infantum*	No
H2-07	WLKGYFRLGVAMESM	E9BT68	71	1.01	7.98	No	99.32	*L. major*, *L. infantum*, *L. mexicana*	No
H2-08	APLMLYISKMVPTAD	E9BT80	376	1.10	3.24	No	93.09	*L. major*, *L. infantum*, *L. mexicana*, *L. braziliensis*, *L. panamensis*	No
H2-09	NTDFVMYVASVPSEG	P23223	194	1.10	8.86	No	90.42	*L. infantum*	No
H2-10	ASDAGYYSALTMAIF	P23223	335	0.89	5.58	No	98.63	None	No
H2-11	LVKYLIPQALQLHTE	P23223	143	0.85	4.67	No	98.72	*L. infantum*	No
H2-12	DILVKYLIPQALQLH	P23223	141	0.74	2.72	No	94.14	*L. infantum*	No
H2-13	SDAGYYSALTMAIFQ	P23223	336	0.70	9.81	No	99.53	None	
H2-14	CNGGLMLQAFEWLLR	Q95WR6	188	1.04	8.42	No	94.48	*L. infantum*	No

^a^100% identity for absolute query cover

^b^Identity threshold in human for amino acids > 12 (80%)

The construct of 397 amino acid residues comprised of the 9 CTL and 14 HTL epitopes, with AAY and GPGPG linkers added in the intra-epitopic positions of CTL and HTL epitopes, respectively. It preceded in N-terminal by TLR4 peptide adjuvant, APPHALS, linked by EAAK linker to the vaccine. The selected rearranged model had the antigenicity score of 0.8 calculated by ANTIGENpro, and 0.74 (bacteria model) and 0.65 (parasite model) by VaxiJen. Furthermore, the construct was found to be non-allergenic for human use. When re-analyzed by the screening tools, all the original CTL and HTL epitopes were found consistent with the pre-screening immunogenicity, cleavage and TAP binding properties in the rearranged model. On the other hand, the arrangement of the construct resulted in generation of only three regions (15-mer overlapping) of IL-10 inducing epitopes and three non-specific CTL epitopes (9-mer) comparable to the potency of target epitopes (Additional file 3: Figure S1).

### IFN-γ epitopes

**Fig. 2:**
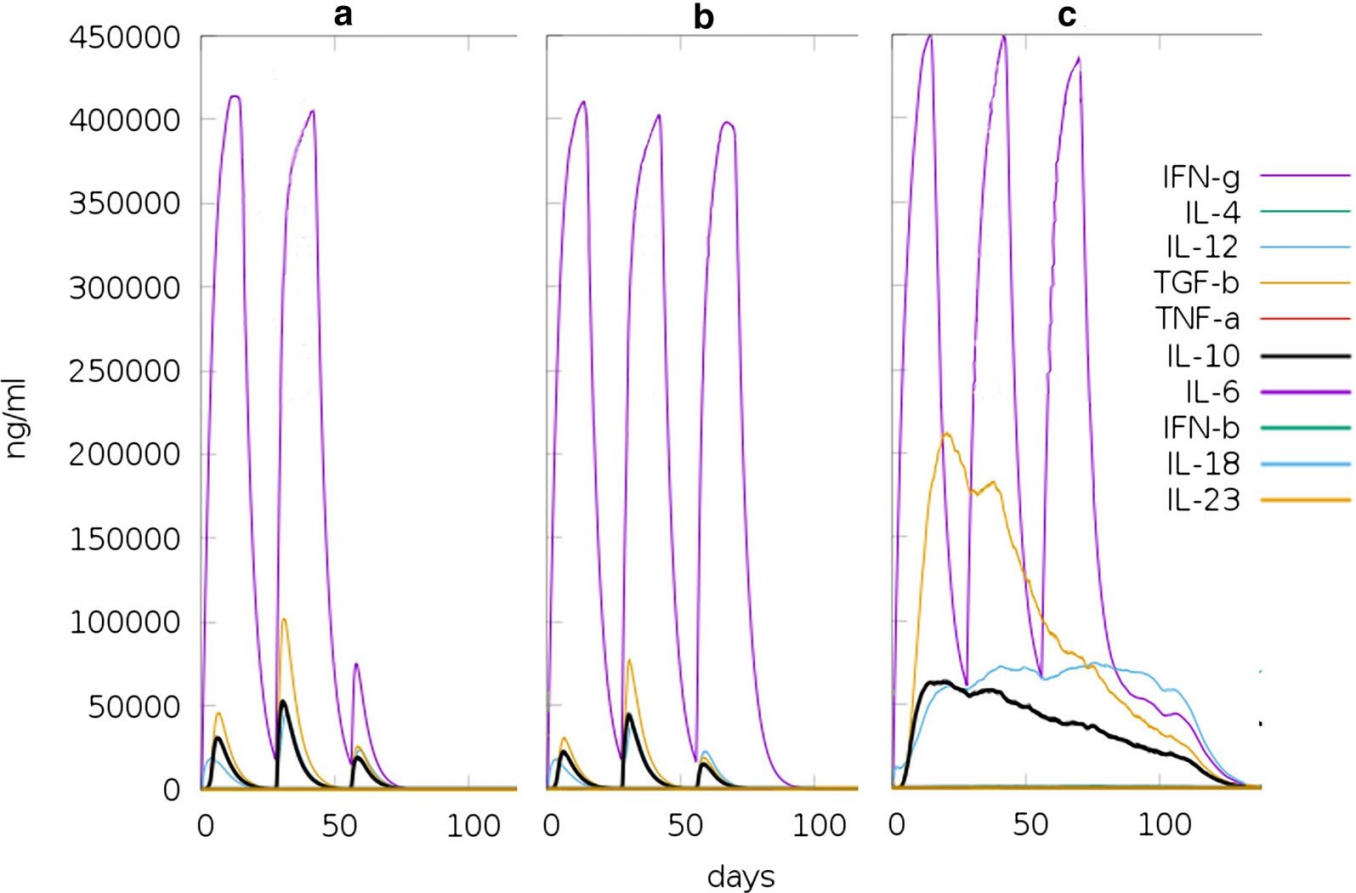
Simulation of cytokine response. Illustration of cytokine induction potential by control peptide 1 (*L. infantum* derived fusion peptide) (**a**), vaccine construct designed in this study (**b**) and control peptide 2 (*L. donovani* GP63 derived peptide) (**c**) by independent simulation of immune response. Hypothetical administration of the peptides was performed in three doses four weeks apart with 1000 units/dose

Prediction on IFN-γ induction capacity revealed a total of 117 epitopes (15-mer) with positive scores. This prediction was consistent with the C-ImmSim simulated immune response in terms of high IFN-γ production after hypothetical immunization (three doses) in a population characterized by a combination of frequent and VL susceptible HLA alleles [59–61]. Since the hypothetical cytokine levels in simulated immune response represent only the outcome of algorithmically set dynamic cellular interactions for a defined time period after antigen priming [37], it was important to evaluate whether the simulation module can respond dynamically to different constructs [45]. Therefore, we simulated immune responses of two additional peptide vaccine candidates: peptide 1 (*L. infantum* derived fusion peptide [46]) and peptide 2 (*L. donovani* GP63 derived peptide [47]), which were experimentally found to exhibit varying cytokine response in comparison to soluble *Leishmania* antigen (SLA). Substantial difference was observed in terms of immunosuppressive IL-10 and TGF-β induction capacity between peptide 1 and peptide 2; however, determination of statistical significance was not possible in the simulation module. Nevertheless, the outcome can be considered consistent with the general trend of *in vitro* immune response (compared to SLA), with peptide 2 being more prominent IL-10 inducer compared to SLA as reported in [47]. In terms of cytokine induction potential, simulation outcome of our designed construct conformed more closely to that of peptide 1, which did not induce IL-10 level higher than that by SLA *in vitro* [46] (Fig. 2).

### Tertiary structure of the chimeric protein and cloning model

Since there was no significant template hit, the choice of 3D model among outputs generated by I-TASSER was based on: (i) cluster size of model replicas; (ii) frequency of model in simulation trajectory; and (iii) C-score. The selected model has the highest C-score of − 1.56 which is close to the I-TASSER recommended score (− 1.5) for accuracy, and has the highest frequency in the top cluster by size. After further refinement of the protein topology, the PROCHECK [62] server returned a G-score of − 0.04, which indicates that the backbone and side chain of the model correspond to high-probability stereochemical conformations. The model scored 1.73 in X-ray resolution scale by MolProbity [63], with no poor rotamers and bad bonds, negligible all atom steric overlaps (0.5%) and an increase in Ramachandran-favored residue number from 79.2% (unrefined) to 92.4% (refined) with a subsequent decrease in outliers (Additional file 4: Figure S2). The vaccine construct has a molecular weight of 42.1 kDa, with a basic nature (isoelectric point: 9.16). The score obtained for instability index was 27.26, which implied the stable nature of the vaccine *in vitro*. The estimated value of aliphatic index was 75.39 which indicated its thermo-stability. The folded structure has a melting temperature of 73.9 °C and folding free energy of − 17.7 kcal/mol at neutral pH in humans. Additionally, this model was found to have substantial solubility with a score of 0.38 in folded state in contrast to the unfolded intrinsic score of − 3.06, which suggests that hydrophobic residues in this model tend to ideally form the stable core leaving hydrophilic residues much on the solvent accessible surface. The half-life of the construct in mammalian reticulocytes was estimated as 4.4 h *in vitro*, compared with 20 h and 10 h in yeasts and *Escherichia coli in vivo*, respectively.

**Fig. 3:**
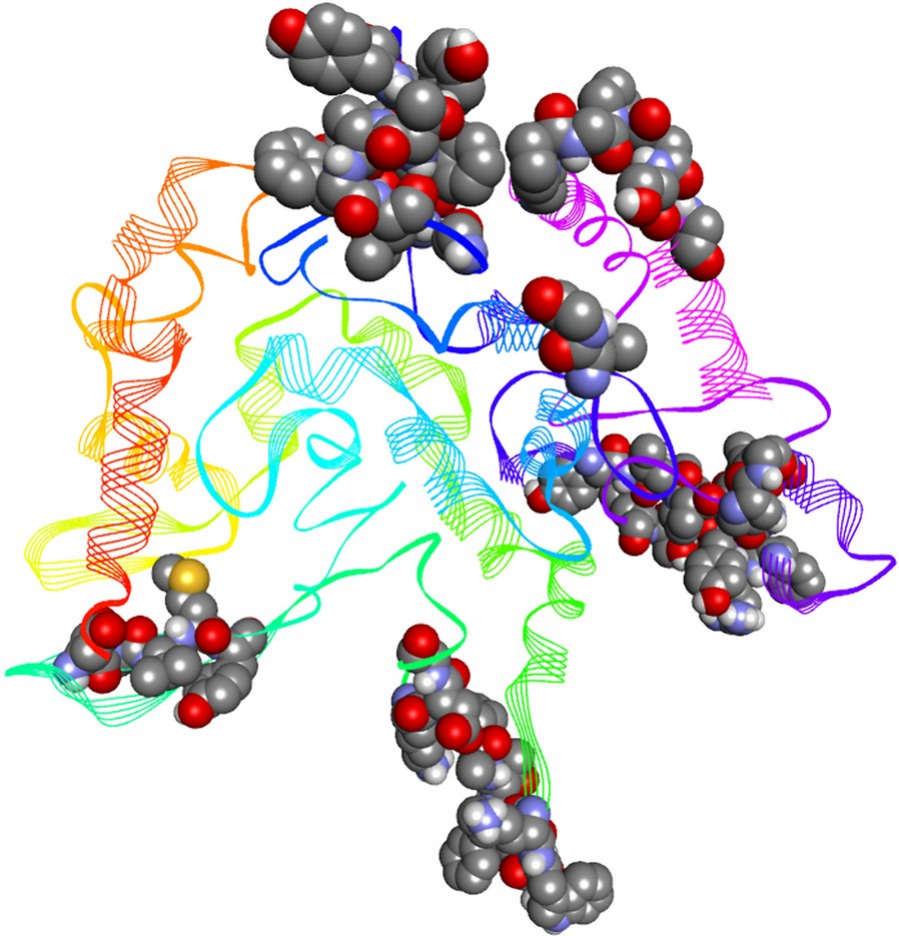
Refined tertiary structure of the chimeric protein. The secondary structure elements consist of helix (34%), sheet (10%), turns (39%) and coil (17%). Residue positions in B cell conformational epitope that overlap linear epitopes are depicted as CPK shapes

In terms of chimera-specific B cell response, Bepipred predicted six B cell epitopes of 8–12 residues in length above the threshold score, while BCPREDS predicted 11 non-overlapping and linear 20-mer B cell epitopes with specificity scores > 0.99. Residues in those linear epitopes accounted for 41% residues of the 08 non-overlapping conformational epitopes (Fig. 3, Additional file 5: Table S1).

This sequence was used to generate *in silico* cloning model for *E. coli* (K12) expression. After optimization of the codon, the codon adaptation index (CAI) value of the chimera was 0.98, while the GC content was 56.09%. For insertion into the *E. coli* pET28a(+) expression vector, two restriction sites for *Xho*I and *Nde*I enzymes were added in the 3ʹ- and 5ʹ-end, respectively, of the vaccine coding strand enclosed by 6-histidine residues at both ends (Additional file 6: Figure S3).

### Molecular docking of vaccine in TLR4

**Fig. 4:**
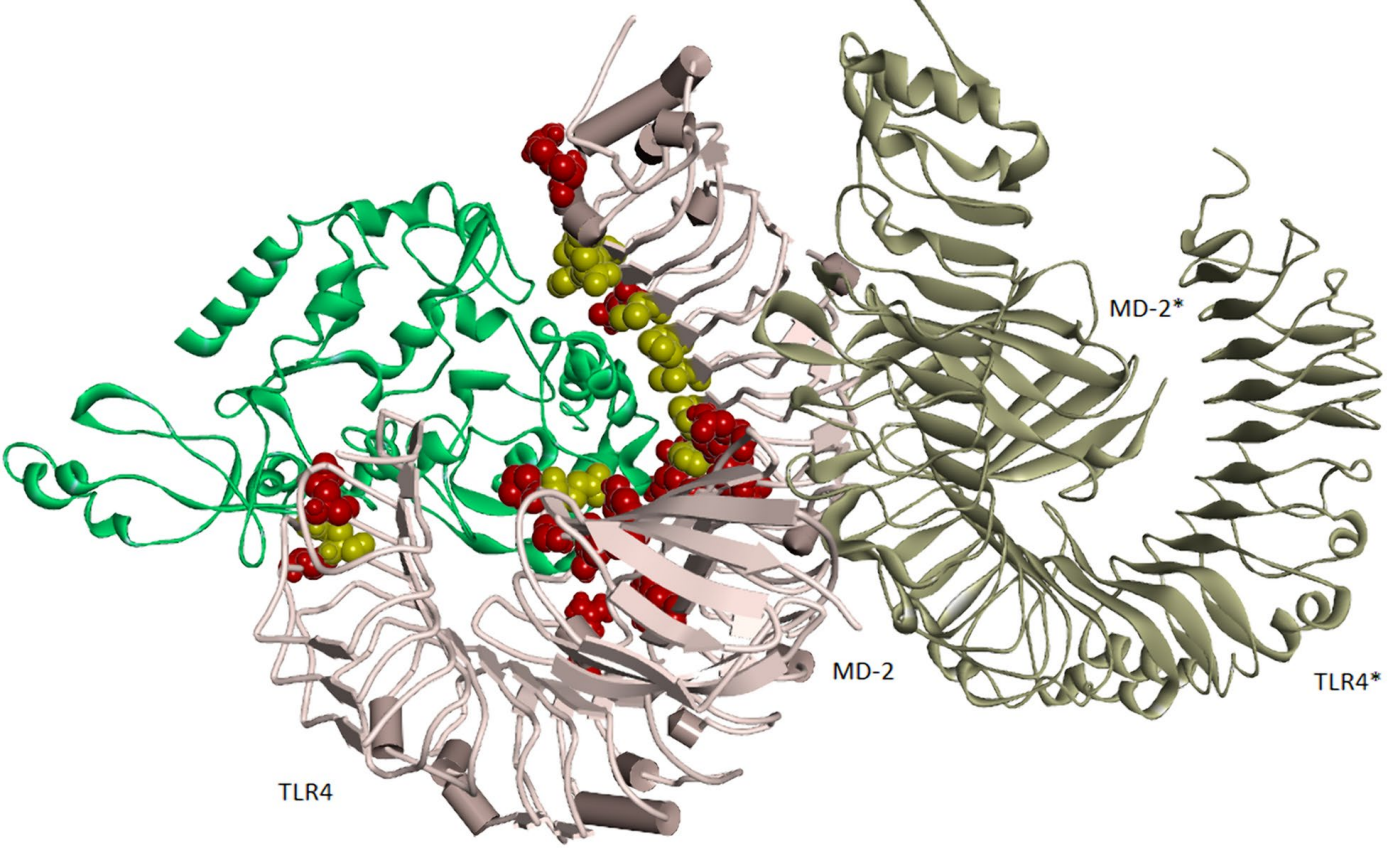
Docked complex of TLR4 with vaccine construct. Accompanying structural monomers include second TLR4 ECD (TLR4*), MD-2 adapter and second adapter (MD-2*). Residual participants of receptor monomers interacting with vaccine (green) are represented by yellow (hydrophobic) and red (hydrogen bond) CPK shapes

Molecular docking of the vaccine construct with TLR4 in ClusPro 2.0 docking server generated 30 models ranked by cluster size of the representative pose. The selected docked complex had the largest cluster size (ClusPro recommended) with second-lowest binding energy score (− 1282.3) among the top ten models. The chimeric construct seemed to occupy partially into the lateral concave surface, but not the convex surface, with strong hydrophobic interactions mostly with the beta-sheet adjacent residues at the C-terminal domain of TLR4 ectodomain (ECD) and also with its adapter protein, MD2, with support of several hydrogen bonds, thus establishing ligand mediated cross-link between TLR4 and MD2 (Fig. 4).

### Molecular dynamics (MD) simulation of vaccine-TLR4 complex

**Fig. 5:**
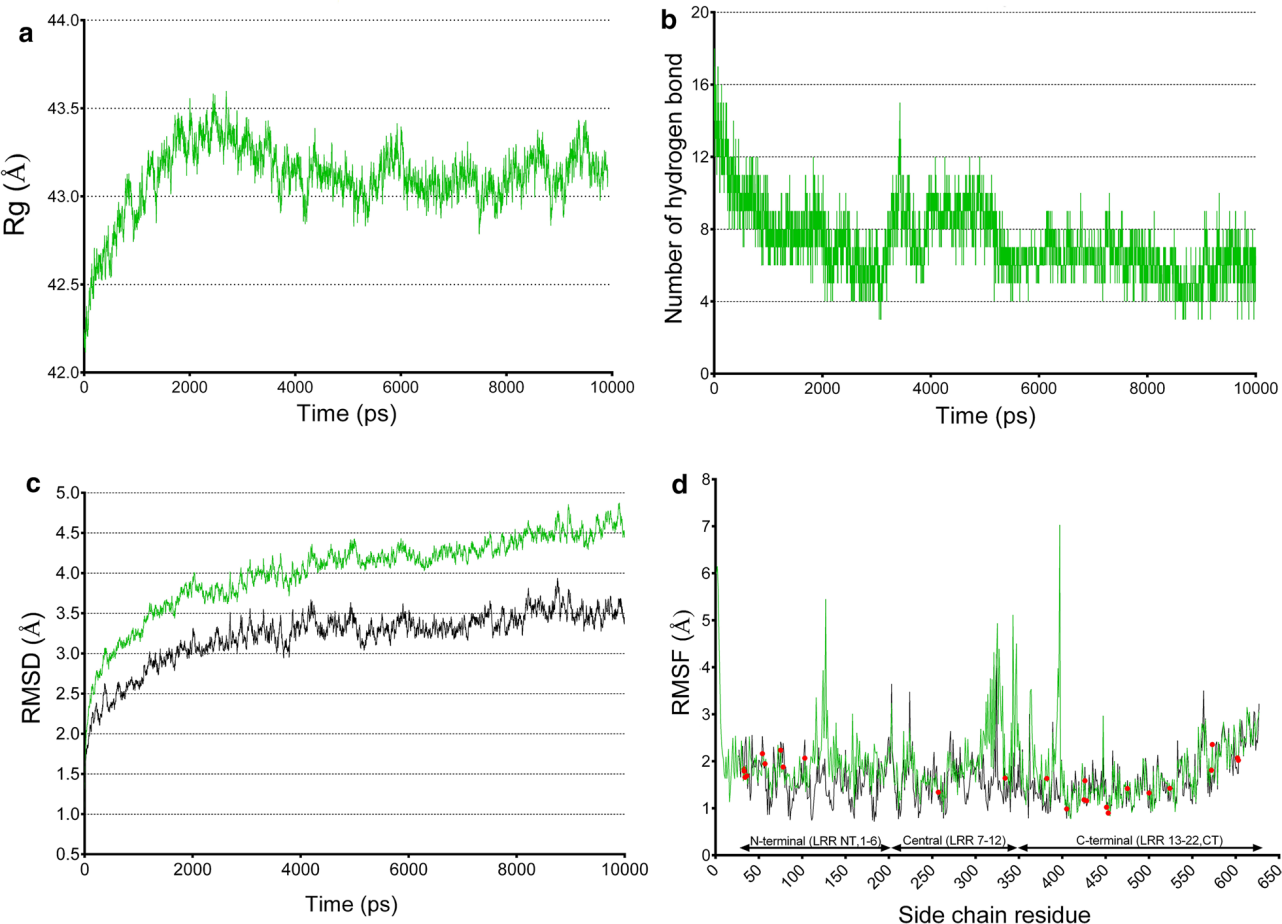
Molecular dynamics simulation of docked complex. For a time duration of 10 ns, plots of the radius of gyration (Rg) (**a**), hydrogen bond (**b**), RMSD of the backbone atoms fitted to complex (green) and ligand-free receptor (black), with respect to initial structure (**c**), and RMSF for side-chain atoms fitted to complex (green) and ligand-free receptor (black) with respect to initial structure of TLR4 ECD (627 aa; divided into three domains of leucine rich repeats or LRR) (**d**). Receptor positions interacting with vaccine (final frame) are represented with red circles

Molecular dynamics simulation of the docked complex was performed by using OPLS_2005 force field. Using the Simulation Quality Analysis tool of the Desmond software, the mean potential energy for the complex was obtained as − 6.4e5 kilocal/mol (Additional file 7: Figure S4). The radius of gyration (Rg) obtained for the docked complex showed that the mean distance in rotating complex from the center of mass is 4.31 nanometers (SD: 0.2 nanometers) about which the model becomes consistent after 4 ns (Fig. 5a). The number of intermolecular hydrogen bond (H-bond) between the side chains of vaccine protein and TLR4 initially fluctuated probably due to solvent effect before matching the trend of Rg in reaching steadiness after 4 ns. This suggests the role of H-bonds in the overall compactness of the complex (Fig. 5b). The trends of Rg and H-bond plots indicate that 6–8 strong H-bonds were persistent over simulation period between vaccine and TLR4, and this might be crucial for stable binding.

The root mean square deviation (RMSD) of the vaccine-TLR4 complex for backbone atoms over the simulation period was 4.0 Å (SD: 0.49 Å), while it was 3.2 Å (SD: 0.35 Å) for ligand-free TLR4 atoms (Fig. 5c), suggesting comparably higher (paired t-test: *P* < 0.0001) RMSD of the complex backbone. The root mean square fluctuation (RMSF) for side-chain atoms of vaccine-bound TLR4 (1.9 Å, SD: 0.7 Å, range: 0.8–7.0 Å) was higher (Wilcoxon matched-pairs test, *P* < 0.0001) than unbound TLR4 (1.6 Å, SD: 0.5 Å, range: 0.7–4.2 Å). The RMSF indicates overall less fluctuations for atoms interacting with vaccine residues, while atoms at vaccine unbound regions of the N-terminal and central domain underwent high fluctuations (Fig. 5d). Although the trends toward reaching convergence were very similar, higher RMSD value of the complex than the vaccine-unbound TLR4 indicates structural mobility in the complex due to vaccine interaction and this is likely attributable to the higher RMSD of vaccine protein along the MD simulation time. In congruence, rearrangement of several bonds between the vaccine and TLR4 was observed between pre-simulation and post-simulation models, while the total number of non-covalent bonds increased from 41 in pre-simulation model to 64 in post-simulation model (not shown). Visualization of the interacting residues also indicates that, in comparison to unbound (and also pre-simulation) structure, the post-simulation bonding rearrangement is coupled with increased number of H-bond at the C-terminal domains between TLR4 and TLR4* (second TLR4 ECD) (Additional file 8: Figure S5). This implies likely chance of positive interactions between the TLR4 monomers in physiological condition following vaccine interaction. Overall, the conformation of vaccine-bound receptor supports structural flexibility, which might be in favor of biological response of the receptor.

### Immune simulation to predict secondary response

Hypothetical administration of three doses of vaccine construct four weeks apart with 1000 unit/dose was performed to simulate the immune response generated by immunization. *In silico* immune simulation plots hinted at antigenic recognition and subsequent response in terms of antibody production, and active as well as memory B cell and T cell generation in the population with a VL susceptible HLA profile after hypothetical immunization. The primary response to the proposed chimera can be characterized by a marked increase in chimera-specific IgM and IgG production. After subsequent doses, a corresponding decrease in antigen concentration indicates gradual increase in memory B cell production with persistence. Furthermore, expansion of CD4+ T lymphocytes with memory development following the initial dose was observed. CD8+ T lymphocytes response was also high for the susceptible population reaching at its peak after the second dose. Repeated exposure of 12 doses, on the other hand, did not seem to cause clonal expansion of any epitope-specific T cells- as indicated by the Simpson’s index D, which is inversely related to diversity (Additional file 9: Figure S6).

## Discussion

Proteomics-driven identification of potential vaccine candidates can be a sound approach for selecting promising antigens, which are elicited against environmental stimuli analogous to host response upon pathogen invasion and are physiologically relevant for pathogens within the host [64]. Availability of pathogen proteome information upon infection of the host can provide opportunities for *in silico* mining of novel vaccine candidates, and this approach has been utilized for *in silico* design of an epitope-based vaccine against *Theileria* parasites of ruminants [65]. For a dimorphic human parasite like *Leishmania*, it is important to target human stage-associated antigenic proteins that are physiologically important for parasites to infect and establish in a new host. In recent years, several studies utilized immunoinformatic approaches of epitope screening in designing epitope-based vaccines. Khatoon et al. [66], Singh et al. [67] and Vakili et al. [68] have previously reported the theoretical potential of *in silico* designed vaccines for visceral leishmaniasis. Notably, in a recent study by Vakili et al. [69], the group further evaluated successfully the immunogenic potential of the multi-epitope vaccine, derived in part from known antigens, by administering the chimeric construct in experimental mice. This suggests that the *in silico* designed vaccines with epitopes derived from appropriate protein targets have the potential to progress toward advanced phases of vaccine development for visceral leishmaniasis. While the *in silico* studies by Khatoon et al. [66] and Singh et al. [67] largely utilized available genomic databases of *L. donovani* to select vaccine targets, Dikhit et al. [11, 70] performed thorough investigations involving *in silico*, *in vitro* and *in vivo* analysis to screen and validate immunogenic epitopes obtained from proteins that are increasingly expressed at the infective parasite stage. Such highly expressed proteins are likely important for physiological and/or infective process of the parasite and thus can be more effective vaccine targets. In this study, we took an approach to select such amastigote proteins in terms of contrasting abundance or specificity (abundant up to the level of detection) from comparative proteome profiles of *L. donovani* promastigotes and amastigotes. Based on the propensity of those proteins for secretion *in vitro* and/or having secretory signal sequence, we further combined immunoinformatic tools to identify candidate antigens that have secretory potential. A comparison of the methodological and outcome features among several studies that have employed *in silico* design and evaluation of epitope-based candidate vaccines against visceral leishmaniasis to date is summarized in Table 4. Overall, our reported vaccine construct was found to be comparable to the earlier exclusively-*in silico* reports in terms of antigenicity, population coverage and receptor interaction. However, experimental studies remain crucial to validate the immunogenic potential of the designed vaccine.

**Table 4:** Summary of related studies on *in silico* design and evaluation of candidate vaccines against visceral leishmaniasis

Target species	Protein source	Principle basis of protein selection	*In silico* screened epitope features	T cell epitope class	Design of vaccine construct	Vaccine evaluation approach	Evaluation features	Theoretical findings	Experimental findings	Reference
*L. donovani*	Published literature on experimental proteomes of promastigotes/ amastigotes	Increased abundance in amastigotes at protein level; experimental secretion; presence of secretion signals; antigenicity scores	MHC-binding affinity; population coverage; IFN-γ epitope; non-IL-10 epitope; non-B cell epitope	MHC I; MHC II	Chimeric: TLR4 adjuvant + MHC I + MHC II (combination selected based on set criteria)	*In silico*	Physicochemical properties; simulation of immune response; structural dynamics of vaccine-receptor complex	Antigenicity score: 0.8; coverage: > 98% global; Th1 type potential response; potentially stable binding to receptor	Not available	This study
*L. donovani*	Screening proteins in GenBank database	Presence of secretion signals	MHC-binding affinity	MHC I; MHC II	Chimeric: TLR4 adjuvant + MHC I + MHC II; disulfide engineering	*In silico*	Physicochemical properties; binding pocket evaluation; structural dynamics of vaccine-receptor complex	Antigenicity score: 0.77; potentially stable binding to receptor	Not available	[66]
*L. donovani*	Complete proteome from TriTryDB database; random proteins	Presence of secretion signal; antigenicity scores	MHC-binding affinity; AAR score; population coverage; cluster analysis	MHC I; MHC II	Ensemble	*In silico*	Physicochemical properties; simulation of immune response; docking of epitope-HLA	Comparable HLA binding affinity of test peptides; coverage: > 99% in endemic area	Not available	[67]
*L. donovani*	Published literature on protein expression	Increased expression in amastigotes	MHC-binding affinity; TAP binding; population coverage; peptide-HLA docking score	MHC I	Ensemble	*In silico, in vitro* and *in vivo*	Population coverage; T cell proliferation; cytokine production; immunization in BALB/c mice	Population coverage: > 92% global	Proliferative CD8+ T cell response; Th1 type cytokine production	[70]
*L. donovani*	Published literature on protein expression	Increased expression in amastigotes	MHC-binding affinity; population coverage; IFN-γ epitope; peptide-HLA docking score	MHC II	Alone or ensemble	*In silico, in vitro* and *in vivo*	Structural dynamics of peptide-HLA complex; cytokine production; T cell proliferation; immunization in BALB/c mice	Potentially stable binding to HLA	Th1 type cytokine production; spleen cell proliferation in mice	[11]
*L. infantum*	Published literature on protein immunogenicity	Experimentally evaluated immunogenic properties; presence of secretion signal	MHC-binding affinity	MHC I; MHC II	Multi-epitope	*In vitro* and *in vivo*	Immunization in BALB/c mice; lymphocytes proliferation assay; cytokine production	Not available	Spleen cell proliferation; Th1 type cytokine production; induction of CD8+ T cells	[10]
*L. infantum*	Previous reports on whole proteome data mining and protein immunogenicity	Predicted antigen from subtractive genomics study; experimentally evaluated immunogenic properties	MHC-binding affinity; IFN-γ epitope	MHC I; MHC II	Chimeric: TLR4 Adjuvant + MHC-I + MHC-II + TLR4 Adjuvant	*In silico*	Physicochemical properties; structural dynamics of vaccine-receptor complex	Antigenicity score: 0.95; potentially stable binding to receptor	Not available (evaluated in a follow-up study)	[68]

Analyzing amastigote secretome through intra-macrophagic studies is considered difficult, while significant difference in secretome between amastigotes and promastigotes is unlikely due to relatively low stage-specific differences in gene expression [27]. However, due to the dynamicity in the relationship between mRNA and protein abundance as *L. donovani* adapts to the amastigote condition, comparative levels of abundance of these secretory proteins can be a more reliable indicator. Hence, our screening approach is relevant within the context. Perhaps, the most studied amastigote-specific vaccine candidate in *L. donovani* happens to be a cellular stress countering abundant surface antigen, A2, which has shown to confer whole or epitope-specific efficacy in multiple immunization models [8, 71]. The vaccine construct reported in the present study comprised of immunogenic T cell-specific epitopes (as predicted immunoinformatically) from 13 amastigote-associated proteins. Five of them are known to associate with virulence in the mammalian host (fructose-1,6-bisphosphatase, putative protein disulfide isomerase, putative lipophosphoglycan biosynthetic protein, leishmanolysin and cysteine protease), while others have putative roles in countering the host-induced stress response (thioredoxin-like protein, glutathione peroxidase, stress-inducible protein STI1 homolog), host-microbicidal activity regulation (proteasome endopeptidase) and protein synthesis (elongation factor 2). Three proteins were uncharacterized according to the proteomic studies. Protein domain and homology (to proteins of other *Leishmania* species) suggest that two of these proteins may potentially play a role in drug resistance phenotype (E9BUW4) and protection from intracellular stress (E9BDB8), while the specific function of alpha/beta hydrolase domain-containing protein (E9BQ40) in amastigotes has not yet been deciphered. On the other hand, lack of reports on experimental evaluation of immunogenicity of several *Leishmania* proteins, which have been included in our set of antigenic proteins is apparent. Among the 13 proteins of current interest, only six (elongation factor 2, proteasome endopeptidase complex, putative protein disulfide isomerase, leishmanolysin, cysteine protease and putative lipophosphoglycan biosynthetic protein) or their species homologs are known to have proven immunoreactive properties (Table 1). Nevertheless, the increased abundance of the unexplored proteins suggests their likely role of pathological/physiological significance in host invasion and/or survival. The antigenicity scores further corroborate to the potentiality of these proteins as antigenic. Immunological evaluation of these amastigote stage-associated proteins may unravel novel *Leishmania* antigens in future.

In the context of functional roles of selected proteins, our designed vaccine has the potential to benefit the host by generating appropriate immune response both in the early and progressive phase of systemic infection. Furthermore, almost all of the epitopes were found in corresponding proteins of *L. infantum*, indicating potential cross-protection against this visceralizing species. Most of the VL cases are reported from the endemic zones of the Indian subcontinent, East Africa and South America. Thus, in designing an epitope-based subunit vaccine, it is important to estimate the fractions of population in the target endemic zones based on HLA genotypic frequencies. The immunogenic non-self CTL epitopes in the vaccine modeled here is estimated to cover 96.8%, 91.7% and 93.9% of the allelic populations of Brazil, India and Sudan, respectively, with experimentally evaluated truly binding affinity [72], while for HTL epitopes, it is almost 100% for each of these populations. The vaccine construct has antigenic properties while it was not found to be an allergen. The structure was found thermodynamically stable and surface-soluble, while the core is hydrophobic, a favorable feature for antigen processing. Vaccine-specific, but not parasite protein-specific humoral response was predicted, and this can be used as a biomarker of vaccine efficacy [46, 73] without eliciting a parasite-specific B cell response. Moreover, the construct structure showed a good binding affinity in previously reported binding cavity of TLR4 [74–77].

The structural interface between TLR4 and the peptide adjuvant (APPHALS) used here has been extensively studied before. The position occupied by the adjuvant peptide in the TLR4-MD2 complex has been suggested to be varying depending on its position in the vaccine model and the canonical activation of the receptor is thought to be mechanized by insertion of peptide adjuvant in MD2 [78]. Since we used already activating but hypo-responsive TLR4-MD2 crystal structure removed of LPS for docking [79], it was not possible to speculate about the agonistic behavior of the bound vaccine. Nevertheless, our docking model is suggestive of non-MD2 (non-canonical) binding of adjuvant linked peptide, in which the vaccine intrinsic segment may have more affinity than the peptide adjuvant for binding to TLR4. The binding interface along with the molecular dynamics (MD) simulation of the docked complex in the solvent system hint at a sufficiently stable cross-link of TLR4 and MD2 with no major bond rearrangement between TLR4 and MD2, and between TLR4* and MD2 heterodimer formations. Although, the simulation time was short, this is reasonable as none of the vaccine residues interacted at crucial MD2-binding sites [74]. On the other hand, H-bond was found to increase between TLR4 ECDs (where vaccine is bound to one TLR4 ECD) in the vaccine-bound form compared to the unbound TLR4, which suggests potential event of positive interactions and movement between the ECDs. Additionally, reduction in electrostatic surface potential at the vaccine-bound TLR4 interface was observed after docking, which was consistent in post-simulation structural interface. Simultaneously, it was observed that a homo-dimer destabilizing His458-His458* repulsion [76] at pre-dock TLR4 was nullified and superseded post-dock by a solvent stable pi-hydrophobic interaction. It is thus possible that a change in the interpolated charge difference between pre-dock and post-dock TLR4 interface could have contributed to the bonding rearrangement between TLR4 ECDs. Notably, this rearrangement also involved participation of other critical histidine (His431, His555) residues at the TLR4-TLR4* interface [80] unlike the unbound structure (Additional file 8: Figure S5). Overall, these events are congruent with non-canonical TLR4 activation model mediated by microbial peptides, metals and cationic lipid nano-carriers, which are suggested to not confer canonical interaction with other monomers but to induce bond rearrangement among receptor monomers upon interaction [74–77]. Although the exact mechanism remains to be elucidated, our observations suggest that the vaccine construct may possess a characteristic peptide feature of a non-canonical TLR4 ligand [81, 82], which may facilitate TLR4-TLR4* dimerization for downstream activation of immune cells. The trends of backbone RMSD, Rg and H-bond of the vaccine-bound complex over the simulation period complied with structural flexibility rather than rigidity of the complex. The RMSF values of the complex side-chain indicate that the higher fluctuations in TLR4 were of those residues, which are vaccine-unbound and located in the solvent exposed loop mostly at or around glycines [83]. Increased residual fluctuation at LRR10-12 and around Gly397 may also be attributed to the mutations introduced at the position 299 and 399 in TLR4 structure (4G8A), as reported in [79], which was used to dock the vaccine protein. Nevertheless, it is unlikely that vaccine interaction would induce dissociation in structural interface of natural TLR4-MD2 since none of the highly fluctuating TLR4 residues had any direct interaction with the vaccine or MD2.

Simulation outcome of hypothetical immunization in VL susceptible HLA alleles (hypothetical heterozygous combination) was consistent with the predicted immunogenicity of the vaccine. Furthermore, we showed that the simulation outcome can be dynamic for different constructs when we used the same criteria in the simulation program and the same HLA profile to test two known vaccine candidates for VL. Importantly, for these peptides, IL-10 production was reported previously as either prominent (peptide-2) or lessened (peptide-1) in comparison to SLA *in vitro*. It is not expected that simulation results will reflect experimental outcomes; however, we observed a general trend of difference in immunosuppressive cytokine (e.g. IL-10) induction potential between the two peptides from the simulation outcome, with peptide-2 having more potent IL-10 induction capacity. Although statistical significance could not be inferred from the simulation plots, the difference seems consistent with the experimental result. Understandably, the predicted epitopes (not shown) in the simulation program did not comply mostly with our target set of epitopes due to the difference in the epitope prediction algorithm [45]. However, when compared to the simulation outcome of the known peptides, the general trend was comparable to both peptides for IFN-γ induction, while TGF-β and IL-10 were predicted to be considerably less pronounced than that by peptide-2. Besides IL-10, TGF-β has potent immunosuppressive properties, enhances disease progression and may prevent cure and protective immunity development against leishmaniasis [84, 85]. Thus, the simulation prediction of higher propensity of the construct to induce a more Th1-polarized response rather than Th2 is consistent with our desired immunogenicity.

Despite the difference in the epitope set, simulation dynamics over time can be extrapolated for the estimated set of epitopes of our construct since it is also comprised of diverse T cell epitopes and vaccine-specific B cell immunogenic regions as predicted by several immunoinformatic tools. It has been proposed previously that the simulation dynamics can be consistent with a realistic immunization process in terms of primary and secondary immune responses [45]. Likewise, clearance of antigen, production of antibody, development and persistence of memory B cells as well as CD4+ T cells over several months were assumed in the simulation outcome. For primary activation and maintenance of CD8+ T cells, CD4+ T cells (both Th1 and Th2 type) [86] are believed to be required [87, 88], where cytokines such as IFN-γ, IL-2 and IL-4 could be involved [89–92]. The simulation outcome suggests chance for expression of high levels of IFN-γ and IL-2, which may potentiate CD8+ T cell expansion. On the other hand, it is unlikely that the vaccine would trigger clonal expansion of epitope-specific T cells since we combined potent epitopes from several amastigote-associated proteins of comparable affinity, and it was consistent with the simulation dynamics for repeated exposure of 12 doses, as indicated by Simpsonʼs index (D). Rather, high level of IL-2 production can be expected for diverse epitope-mediated immune response functional over long time in vaccine-mediated immunity.

Experimental validation is utmost to prove this computational work. Next phases of the reverse vaccinology approach would ideally involve assessing the recombinant immunogenic protein expressed in the *E. coli* (strain K12) system as proposed here, *in vitro* stimulation of peripheral blood mononuclear cells from active VL patients as well as healthy endemic people for cytokine production, and evaluation in challenge models. While a multi-epitope vaccine molecule generated by using a reverse vaccinology approach can induce specific responses in *in vivo* and *in vitro* assays, a single recombinant molecule can also reduce the cost of production [93, 94]. The *in silico* designed vaccine reported here confers substantial immunogenic potential to be considered for *in vitro* experimental evaluation in the next phase of the study.

## Conclusions

Screening and design of large-scale subunit/peptide vaccine candidates can be facilitated by a reverse vaccinology approach prior to experimental validation. This modelling study took a systematic approach to apply a series of immunoinformatic tools to extract T cell-specific epitopes from MS-driven human stage-associated *L. donovani* proteins with secretory potential, and design a subunit vaccine with a broad population coverage. Development of such a prophylactic vaccine for VL may complement therapeutic strategies against active infections as well. Overall, collective approaches of *in silico*, *in vitro* and *in vivo* investigations are utmost to develop a universal subunit vaccine against human VL.

## Supplementary information

**Additional file 1: Text S1.** Methodological details. Reasoning and description of tools used in the study.

**Additional file 2: Data S1.** Database of screened proteins from proteomic studies, and of MHC I and MHC II molecules considered for epitope prediction.

**Additional file 3: Figure S1.** Proposed vaccine construct. The peptide adjuvant precedes CTL (H1) and HTL (H2) epitopes. Non-specific CTL epitopes and IL-10 inducing HTL epitopes are underlined in black and blue, respectively.

**Additional file 4: Figure S2.** Ramachandran plot of the refined structure of the vaccine construct.

**Additional file 5: Table S1.** Account of conformational epitopes in the vaccine construct.

**Additional file 6: Figure S3.***In silico* restriction cloning of vaccine construct. The vaccine coding region is colored red and the green arrow underneath indicates direction of transcription of open reading frame.

**Additional file 7: Figure S4.** Simulation quality analysis. **a** The plot of thermodynamic properties as a function of simulation time over a period of 10 ns. **b** Tabular summary of thermodynamic properties. *Abbreviations*: T.E, total energy; P.E, potential energy.

**Additional file 8: Figure S5.** Bond re-arrangement at the C-terminal domain of TLR4-TLR4* junction. **a** Vaccine-unbound interface. **b** Vaccine-bound interface (post-simulation). Hydrogen bonds are represented as conventional bonds. Interacting surface of TLR4 (encircled) highlights residual charges ranging from positive (blue) to negative (magenta). Inset pictures (right) indicate a transition in the mode of interaction between His458 and His458*.

**Additional file 9: Figure S6.** Simulated immune response following hypothetical immunization. **a** Immunoglobulin production. **b** B cell response and memory development. **c** TH (helper T) cell population per state. **d** TC (cytotoxic T) cell population per state. **e** Cytokine levels at regular dose intervals for 12 doses. The inset plot indicates the level of leukocyte growth factor (IL-2) and the potential for clonal expansion (D) after each dose.

## Data Availability

Data supporting the conclusions of this article are included within the article and its additional files.

## Abbreviations

VL: visceral leishmaniasis
KEP: Kala-azar Elimination Programme
MS: mass spectrometry
CTL: cytotoxic T-lymphocyte
HTL: helper T-lymphocyte
HLA: human leukocyte antigen
TPC: theoretical population coverage
CAI: codon adaptation index
ECD: ectodomain
MD: molecular dynamics
Rg: radius of gyration
RMSD: root mean square deviation
RMSF: root mean square fluctuation
