# Supplementary material for: An immunoinformatic approach driven by experimental proteomics: in silico design of a subunit candidate vaccine targeting secretory proteins of Leishmania donovani amastigotes
Source: Parasit Vectors. 2020 Apr 15;13:196. doi: 10.1186/s13071-020-04064-8 (PMC7160903; doi:10.1186/s13071-020-04064-8)
Supplement: Supplementary file 1 — Additional file 1: Text S1. Methodological details. Reasoning and description of tools used in the study. [file 13071_2020_4064_MOESM1_ESM.docx]

**Additional file 1: Text S1. Methodological details**

Screening of L. donovani secretory antigens associated with amastigote stage

A query was performed using NCBI MeSH terms “Leishmania donovani” and “proteome” in the Pubmed literature database on proteomic studies of *L. donovani* exclusive to the search term. Research articles that quantitatively evaluated MS-driven proteomic abundance of amastigotes were selected to create a local database of proteins. By using the provided IDs, which are either for genes or proteins, corresponding Uniprot protein accessions were retrieved, either directly from UniprotKB database (<https://www.uniprot.org/help/uniprotkb>), or via query in NCBI database followed by BLASTP search (https://blast.ncbi.nlm.nih.gov/Blast.cgi). Similar protein sequences of *L. donovani* with at least 90% identity for total query length within UniRef cluster [[1](#_ENREF_1)] were retrieved. Similarly, Uniprot accession IDs and sequences of secretome of *L. donovani* were retrieved. A local search was performed to match the screened amastigote proteins against the secretome proteins using ViroBLAST with a cut-off value of 90% for both query cover and identity. Subsequently, amastigote proteins having classical or non-classical secretory signal sequences with no more than one transmembrane helix were predicted by SignalP v4.0, SecretomeP and TMHMM web tools. All the cross-matched and predicted secretory proteins were then subjected for antigenicity prediction by ANTIGENpro and VaxiJen. The overall accuracy of ANTIGENpro has been reported to be more than 75% with a prediction threshold of 0.5, while accuracies of VaxiJen in internal and external validation lies in 70-89%, with highest accuracy observed at a threshold of 0.5 (default threshold for parasite model: 0.4). Therefore, proteins that have antigenic probability estimation of ≥ 0.5 by both tools were selected for next steps.

Cytotoxic T-lymphocyte (CTL) epitope prediction and immunogenicity assessment

CTL epitopes have the abilities to elicit cell-mediated immunity. MHC class I molecules that bind short peptides majorly at the peptide anchor positions are highly polymorphic, albeit can be clustered into sets of molecules (supertypes) that bind largely overlapping peptide repertoires [[2](#_ENREF_2)]. By using default parameters of NetCTL v1.2 server (<http://www.cbs.dtu.dk/services/NetCTL/>), CTL epitopes (9-mer) were primarily screened for a threshold score of ≥ 0.75 against all the 12 MHC class I supertypes. At this threshold, NetCTL prediction has a sensitivity of 80% and specificity of 97%. Epitopes covering ≥ 4 supertypes were then treated in both VaxiJen (cut-off: 0.7) and IEDB (<http://tools.iedb.org/main/tcell/>) MHC class I immunogenicity prediction module for screening of highly immunogenic epitopes, followed by filtration using TAPpred to maximize the chance of selecting true TAP transport binder. 9-mer epitopes that may induce IL-10 as predicted by IL-10Pred (default parameters), were excluded. To avoid sequence homology to human self-epitopes, BLASTP search was carried out against human (taxid: 9606) non-redundant protein database. Epitopes with identical stretches greater than seven amino acids in length were discarded. CTL epitopes having B-cell epitope stretch of more than 7 amino acid, as predicted by Bepipred linear epitope prediction module (<http://tools.iedb.org/bcell/>), were removed from the epitope set. The vital step in the development of a peptide-based vaccine is the selection of cross-presented epitopes with broader population coverage. As an evading mechanism, *Leishmania* inhibits “antigen cross-priming” through direct cleavage of the SNARE VAMP8 [[3](#_ENREF_3)]. Since it has been well documented about the vital role of cross-priming in *Leishmania* vaccine [[4](#_ENREF_4)], selection of multiple epitopes with different HLA binding specificities will increase the target population. HLA cross-presentation of the epitopes was analyzed by IEDB recommended module for binding to a frequently occurring set of 70 HLA-A, B and C human alleles (see: Additional file 2). Alleles within 1.0 percentile rank of binding probability were subjected for estimation of theoretical population coverage (TPC) using population tool of IEDB. Promiscuous epitopes each having more than 40% world TPC were short-listed as candidates for vaccine construction.

Helper T-lymphocyte (HTL) epitope prediction and immunogenicity assessment

Helper T- cell response, mainly of Th1 type is most likely to be a crucial part of the prophylactic vaccine against human visceral leishmaniasis. HTL epitopes of 15-mer length were preliminary screened using IEDB recommended MHC class II consensus prediction module with a percentile rank of <1.0 (IEDB recommended value: 10 percentile) for a complete set of human MHC II molecules derived from NetMHCII [[5](#_ENREF_5)] v2.3 server: 25 HLA-DR, 20 HLA-DQ and 9 HLA-DP alleles (see: Additional file 2). Since, our target was to cover both affinity and broad coverage in human, epitopes were preliminary narrowed down by selection of only those epitopes that bind to ≥ 3 different receptor alleles as predicted by consensus method, thus excluding those that may have lower coverage. 15-mer epitopes that may induce IL-10 as predicted by IL-10Pred (default parameters), were excluded, and immunogenically more potent peptides were screened using 7-allele method [[6](#_ENREF_6)] (percentile rank threshold of 10%), and VaxiJen (cut-off: 0.7). BLASTP search was carried out against human (taxid: 9606) non-redundant protein database with threshold of < 80% for query coverage and identity to rule out self epitopes. B-cell epitopes were screened by Bepipred and were excluded as mentioned above. For selection of cross-presented alleles by NetMHCIIpan v3.1 module of IEDB, a threshold of <100nM (IEDB recommended value: 1µM) for binding affinity (IC_50_) was considered. Consensus epitopes that are preferentially cross-presented to alleles with >90% world TPC were shortlisted as candidates for vaccine construction.

Epitope conservancy in other Leishmania species

All the individual HTL and CTL epitopes were used as input of BLASTP search tool against *Leishmania* (taxid: 5658) RefSeq protein database of NCBI to predict conservation of epitopes in representative proteins of other *Leishmania* species causing clinical pathologies.

Multi-epitope subunit vaccine construct and in silico cloning

A vaccine sequence was constructed using the selected CTL and HTL epitopes, which were linked together by AAY and GPGPG linkers, respectively, which may produce better immunogenicity. This sequence was preceded by a synthetic peptide adjuvant (sequence: APPHALS) linked by EAAAK linker. This adjuvant was chosen because of its TLR4 agonizing activity [[7](#_ENREF_7)], since TLR4 mediated immune modulation is well established as a crucial mechanism of both immune evasion exploited by *L. donovani* and anti-leishmania therapeutic activity. Finally, in order to choose the best vaccine construct, a number of intra-epitope combinations within CTL and HTL epitopes, respectively, were made randomly. The selection was made such that the chosen product - 1) has antigenicity score of at least 0.75 in ANTIGENpro and 0.65 in VaxiJen models, based on previous reports of vaccine models [[8](#_ENREF_8), [9](#_ENREF_9)]), 2) is non-allergenic for human use as predicted by AlgPred and AllerTOP v2.0), 3) generates none or least number of new (non-specific) CTL epitopes and IL-10 inducing HTL epitopes due to the arrangement. Further, the selected construct was subjected to IFN-γ induction capacity as it plays important role in vaccine mediated immunity by stimulating macrophages and natural killer cells along with an increased response to MHC antigens. IFN-γ epitopes were predicted using IFNepitope by motif and support vector machine (SVM) hybrid approach against a model based on IFN-γ inducing and non-inducing MHC-II binders.

Tertiary structure prediction, refinement and validation

Structural stability of a recombinant vaccine protein is crucial for efficient presentation of antigenic peptides on MHC after internalization [[10](#_ENREF_10)]. The tertiary structure of vaccine construct was obtained by utilizing default parameters of I-TASSER (https://zhanglab.ccmb.med.umich.edu/I-TASSER/), which generates full-length atomic structural models from multiple threading alignments and iterative template fragment assembly simulations followed by atomic-level structure refinement. The quality of I-TASSER models are estimated by a confidence score (C-score) that combines threading significance and simulation convergence scores. First output model usually has the highest C-score, however, for hard targets, where threading does not have significant template hits, the first model is not necessarily the best model [[11](#_ENREF_11)]. Moreover, a C-score cut-off > -1.5 with correct topology in I-TASSER can give very accurate prediction with error rate less than 0.1. To further improve protein topology, the selected model was subjected to several cycles of energy minimization using the YASARA force field (http://www.yasara.org) followed by deployment in GalaxyRefine web server, which reconstructs the protein side chain followed by repacking and relaxation through molecular dynamics simulation. The refined structure was evaluated in 1) PROCHECK, which assess the stereochemical quality of an unknown structure by G-factor (threshold >-0.5), and 2) MolProbity, which combines the all-atom contact analysis, clash-score, rotamer, and Ramachandran evaluations into a single score normalized to be on the same scale as X-ray resolution.

Physicochemical properties and chimera specific humoral response

ProtParam web tool was used to estimate molecular weight (kDa), estimated half-life, theoretical pI and aliphatic index of the linear construct. Vaccine solubility in unfolded and folded (structure-corrected) state was compared (+1: highly soluble, -1: poorly soluble) by CamSol method, because it is common for stably folded globular proteins to possess large poorly soluble regions in their intrinsic profiles, which usually form the hydrophobic core of the folded state. Further, thermo-dynamic stability of the folded protein was evaluated by SCooP. Scoop predicts the Gibbs-Helmholtz equation associated to the folding transition of a protein structure. The folding free energy is negative in the temperature region contained between the cold and hot (un)folding transitions, where the folded conformation is preferred with respect to the unfolded state. The calculations are done for respective host temperature at a pH near 7.0.

Although our molecule was built with only T cell epitopes and B cell epitope region were excluded, it has been previously reported that humoral response specific against the recombinant chimera confers advantages for use as biomarker of efficacy/immunogenicity induced by the chimeric vaccine and can be correlated with protective immunity against *Leishmania* parasites after immunization [[12](#_ENREF_12), [13](#_ENREF_13)]. Bepipred and BCPREDS server were used to predict non-overlapping linear B-cell epitopes. Bepipred threshold was as mentioned before, while BCPREDS specificity threshold was set at 90%. Conformational epitopes were predicted from the 3D structure of vaccine construct using ElliPro server with a cut-off value of 0.7.

Molecular docking of vaccine with TLR4/MD2 complex

Docking study is important to evaluate the binding interaction between immune cells and the vaccine protein through surface receptors, such as TLR, for proper capture and internalization. As we have incorporated a peptide adjuvant as well, it is also important to assess the interaction of the adjuvant molecule with receptor for accelerating immune response generation. A PDB structure TLR4/MD2/LPS complex (4G8A) was obtained from RCSB Protein Data Bank (https://www.rcsb.org/), cleaned of water molecules and ligands by PyMol (The PyMOL Molecular Graphics System v1.7.4.5, Schrödinger, LLC.), and minimized of energy by using YASARA force field. Using refined vaccine protein as ligand, docking into TLR4/MD-2 complex was performed by ClusPro 2.0: protein-protein docking server for selecting energetically favored most probable binding conformation. The extracellular N-terminal ligand recognition ectodomain (ECD) of TLR4 contains 21 leucine reach repeats (LRR) that adopt a solenoid structure forced into a curved configuration, resulting in a concave and a convex surface. Unlike most known TLR structures, TLR4 uses the concave beta-sheet surface for ligand binding, since it allocates here its adaptor protein [[14](#_ENREF_14)], MD2, which is exclusively needed for the activation of TLR4 upon ligand interaction regardless of canonical [[15](#_ENREF_15)] or non-canonical ligand binding site specificity [[16-18](#_ENREF_16)]. After docking, all structure-complexes were visualized in BIOVIA Discovery Studio Visualization software v4.1 (http://www.accelrys.com) to evaluate binding site and bonding interactions.

Molecular dynamics (MD) simulation of vaccine-TLR4 docked complex

To determine the stability of vaccine-TLR4 complex derived as ClusPro output, MD simulation was carried out using Desmond v5.3 (Schrödinger, LLC, New York, NY, 2018). The complex was first refined and minimized with the Protein Preparation Wizard of Maestro v11.5 (Schrödinger, LLC, New York, NY, 2018) followed by solvent system generation with predefined TIP3P water model in an orthorhombic periodic boundary box, and addition of counter ions to neutralize system charge as well as salt (Na^+^Cl^-^ concentration 0.15M). The default Desmond protocol involved relaxation of the system in the initiation phase followed by equilibration, which includes several short MD simulations of 100ps, 12ps and 24ps simulation under NVT and NPT ensembles with and without restraints on solute heavy atoms. Finally, MD simulation was executed for the production run time duration of 10ns under the NPT ensemble with a time step of 2fs at a constant temperature of 300 K and pressure (bar) of 1.01325 using Nose-Hoover thermostat and Martyna-Tobias-Klein barostat methods. The OPLS_2005 force field [[19](#_ENREF_19)] parameters were used throughout the simulation protocol. The coordinates of all atoms in the system were saved every 2ps of simulation period. The radius of gyration (Rg), root mean square deviation (RMSD) for backbone and root mean square fluctuation (RMSF) for side chain was calculated from the trajectory files by utilizing data generated in Simulation Interaction Diagram and Simulation Event Analysis modules. Graphpad Prism software v.7 was used to produce final figures.

Simulation of vaccine induced immune response

To further characterize the immunogenic potential of vaccine at cellular level, C-ImmSim server (http://150.146.2.1/C-IMMSIM/index.php) was used to simulate immune response following hypothetical injection of vaccine. C-ImmSim model combines immune epitope prediction methods and machine learning techniques for measuring antigen-immune cell interactions in its agent-based simulation process. It incorporates several biological theories related to cellular behavior and represents one cubic millimeter of a lymph node of a vertebrate animal or, one milliliter of peripheral blood as a two-dimensional triangular lattice [[20](#_ENREF_20)].

The purpose of using this module was to predict on active and memory cell generation, and cytokine response. First, evaluation of dynamicity in immunogenic potential prediction was performed by simulating immune response of two experimentally evaluated vaccine candidates differing in construct and *in vitro* performance: peptide 1- *L. infantum* derived fusion peptide (LQTVNMAVRVLYQPNVENLYHIYRHIGVNYAETVLPSLINEIIRAVIAQFNASDLLIKRPEVSHRIGVMLAERAKRFNIDITDVSITQMSFGQAEQEKQAAILLAQGEAEAATLVGNAVKRMPAFLELRGLEAARTIAKTLRDHGNGRYYLDSDSLYVNVKDLKIDHSGAMSQAKYKEAIAYYTKAIELQPDNAVFFANRAAAHTHLKDYNNAIIDCERAIIINPEYSKSYSRLGTALFYQENYSRAVDAFTKAKRAEEKAKATALSTGGGMGGFPGMGGFPEMGGMPDMSQFANMMSNPQFMETAQRMMQNPEFSNLVANMASKFSQGLRVVFEFLCPANPTKARLRVERIYWIAWKQFLYGFFMSGVGTMLMLVGIGCTAQYCVEKARGAGVMLGAFLLCVPGYYSLFVLYMYV), which produced significantly higher level of IFN-γ and low level of IL-10 than soluble *Leishmania* antigen (SLA) following *in vitro* stimulation of cells derived from VL patients [[12](#_ENREF_12)], and peptide 2- *L. donovani* GP63 derived peptide (peptide 2: STHRHRSVAARLVRLAAAGAAVIA), which produced significantly higher level of IL-10 than SLA following *in vitro* stimulation of whole blood of exposed subjects [[21](#_ENREF_21)]. For simulation input against all the three peptide sequences, HLA alleles A0201, A2602, B0702, B5701, DRB1_1301, and DRB1_1404 were selected on the basis of population frequency and genetic susceptibility for VL development [[22-24](#_ENREF_22)]. Simulation time steps was set to 1000 (each step is equivalent to 8 hours) with default dose of injection performed three times four weeks apart (corresponding time steps of injection at 1, 84, and 168). Furthermore, after a repeated exposure of the vaccine antigen for 12 times with intervals of four weeks each, clonal expansion was checked using the Simpson index, D (a measure of diversity). C-ImmSim produced figure legends were edited manually to produce final figures.

In silico cloning

In order to attain optimum expression of the vaccine protein, the primary codon sequence of vaccine protein was adapted for *E. coli* (strain K12) expression system by Codon Adaptation Tool (JCAT), which provides codon adaptation index (CAI) as output measure. By using the restriction cloning module of SnapGene software (GSL Biotech), the adapted codon sequence was cloned into *E. coli* pET28a(+) expression vector.

References

1. Suzek BE, Wang Y, Huang H, McGarvey PB, Wu CH: **UniRef clusters: a comprehensive and scalable alternative for improving sequence similarity searches**. *Bioinformatics (Oxford, England)* 2015, **31**(6):926-932.

2. Sidney J, Peters B, Frahm N, Brander C, Sette A: **HLA class I supertypes: a revised and updated classification**. *BMC Immunology* 2008, **9**(1):1.

3. Matheoud D, Moradin N, Bellemare-Pelletier A, Shio MT, Hong WJ, Olivier M, Gagnon E, Desjardins M, Descoteaux A: **Leishmania evades host immunity by inhibiting antigen cross-presentation through direct cleavage of the SNARE VAMP8**. *Cell host & microbe* 2013, **14**(1):15-25.

4. Bertholet S, Debrabant A, Afrin F, Caler E, Mendez S, Tabbara KS, Belkaid Y, Sacks DL: **Antigen requirements for efficient priming of CD8+ T cells by Leishmania major-infected dendritic cells**. *Infection and immunity* 2005, **73**(10):6620-6628.

5. Jensen KK, Andreatta M, Marcatili P, Buus S, Greenbaum JA, Yan Z, Sette A, Peters B, Nielsen M: **Improved methods for predicting peptide binding affinity to MHC class II molecules**. *Immunology* 2018, **154**(3):394-406.

6. Paul S, Lindestam Arlehamn CS, Scriba TJ, Dillon MB, Oseroff C, Hinz D, McKinney DM, Carrasco Pro S, Sidney J, Peters B *et al*: **Development and validation of a broad scheme for prediction of HLA class II restricted T cell epitopes**. *Journal of immunological methods* 2015, **422**:28-34.

7. Shanmugam A, Rajoria S, George AL, Mittelman A, Suriano R, Tiwari RK: **Synthetic Toll like receptor-4 (TLR-4) agonist peptides as a novel class of adjuvants**. *PloS one* 2012, **7**(2):e30839.

8. Ali M, Pandey RK, Khatoon N, Narula A, Mishra A, Prajapati VK: **Exploring dengue genome to construct a multi-epitope based subunit vaccine by utilizing immunoinformatics approach to battle against dengue infection**. *Scientific reports* 2017, **7**(1):9232.

9. Pandey RK, Bhatt TK, Prajapati VK: **Novel Immunoinformatics Approaches to Design Multi-epitope Subunit Vaccine for Malaria by Investigating Anopheles Salivary Protein**. *Scientific reports* 2018, **8**(1):1125.

10. Scheiblhofer S, Laimer J, Machado Y, Weiss R, Thalhamer J: **Influence of protein fold stability on immunogenicity and its implications for vaccine design**. *Expert review of vaccines* 2017, **16**(5):479-489.

11. Roy A, Xu D, Poisson J, Zhang Y: **A protocol for computer-based protein structure and function prediction**. *Journal of visualized experiments : JoVE* 2011(57):e3259.

12. Dias DS, Ribeiro PAF, Martins VT, Lage DP, Costa LE, Chavez-Fumagalli MA, Ramos FF, Santos TTO, Ludolf F, Oliveira JS *et al*: **Vaccination with a CD4(+) and CD8(+) T-cell epitopes-based recombinant chimeric protein derived from Leishmania infantum proteins confers protective immunity against visceral leishmaniasis**. *Translational research : the journal of laboratory and clinical medicine* 2018, **200**:18-34.

13. Martins VT, Duarte MC, Lage DP, Costa LE, Carvalho AM, Mendes TA, Roatt BM, Menezes-Souza D, Soto M, Coelho EA: **A recombinant chimeric protein composed of human and mice-specific CD4(+) and CD8(+) T-cell epitopes protects against visceral leishmaniasis**. *Parasite immunology* 2017, **39**(1).

14. Botos I, Segal DM, Davies DR: **The structural biology of Toll-like receptors**. *Structure (London, England : 1993)* 2011, **19**(4):447-459.

15. Park BS, Song DH, Kim HM, Choi BS, Lee H, Lee JO: **The structural basis of lipopolysaccharide recognition by the TLR4-MD-2 complex**. *Nature* 2009, **458**(7242):1191-1195.

16. Oblak A, Pohar J, Jerala R: **MD-2 determinants of nickel and cobalt-mediated activation of human TLR4**. *PloS one* 2015, **10**(3):e0120583.

17. Schmidt M, Raghavan B, Muller V, Vogl T, Fejer G, Tchaptchet S, Keck S, Kalis C, Nielsen PJ, Galanos C *et al*: **Crucial role for human Toll-like receptor 4 in the development of contact allergy to nickel**. *Nature immunology* 2010, **11**(9):814-819.

18. Lonez C, Irvine KL, Pizzuto M, Schmidt BI, Gay NJ, Ruysschaert JM, Gangloff M, Bryant CE: **Critical residues involved in Toll-like receptor 4 activation by cationic lipid nanocarriers are not located at the lipopolysaccharide-binding interface**. *Cellular and molecular life sciences : CMLS* 2015, **72**(20):3971-3982.

19. Shivakumar D, Williams J, Wu Y, Damm W, Shelley J, Sherman W: **Prediction of Absolute Solvation Free Energies using Molecular Dynamics Free Energy Perturbation and the OPLS Force Field**. *Journal of chemical theory and computation* 2010, **6**(5):1509-1519.

20. Rapin N, Lund O, Bernaschi M, Castiglione F: **Computational immunology meets bioinformatics: the use of prediction tools for molecular binding in the simulation of the immune system**. *PloS one* 2010, **5**(4):e9862.

21. Elfaki MEE, Khalil EAG, De Groot AS, Musa AM, Gutierrez A, Younis BM, Salih KAM, El-Hassan AM: **Immunogenicity and immune modulatory effects of in silico predicted L. donovani candidate peptide vaccines**. *Hum Vaccin Immunother* 2012, **8**(12):1769-1774.

22. Singh T, Fakiola M, Oommen J, Singh AP, Singh AK, Smith N, Chakravarty J, Sundar S, Blackwell JM: **Epitope-Binding Characteristics for Risk versus Protective DRB1 Alleles for Visceral Leishmaniasis**. *The Journal of Immunology* 2018, **200**(8):2727-2737.

23. Faghiri Z, Tabei SZ, Taheri F: **Study of the association of HLA class I antigens with kala-azar**. *Human heredity* 1995, **45**(5):258-261.

24. Kaye PM, Aebischer T: **Visceral leishmaniasis: immunology and prospects for a vaccine**. *Clinical microbiology and infection : the official publication of the European Society of Clinical Microbiology and Infectious Diseases* 2011, **17**(10):1462-1470.
