# Supplementary figures and images for: An immunoinformatic approach driven by experimental proteomics: in silico design of a subunit candidate vaccine targeting secretory proteins of Leishmania donovani amastigotes
Source: Parasit Vectors. 2020 Apr 15;13:196. doi: 10.1186/s13071-020-04064-8 (PMC7160903; doi:10.1186/s13071-020-04064-8)

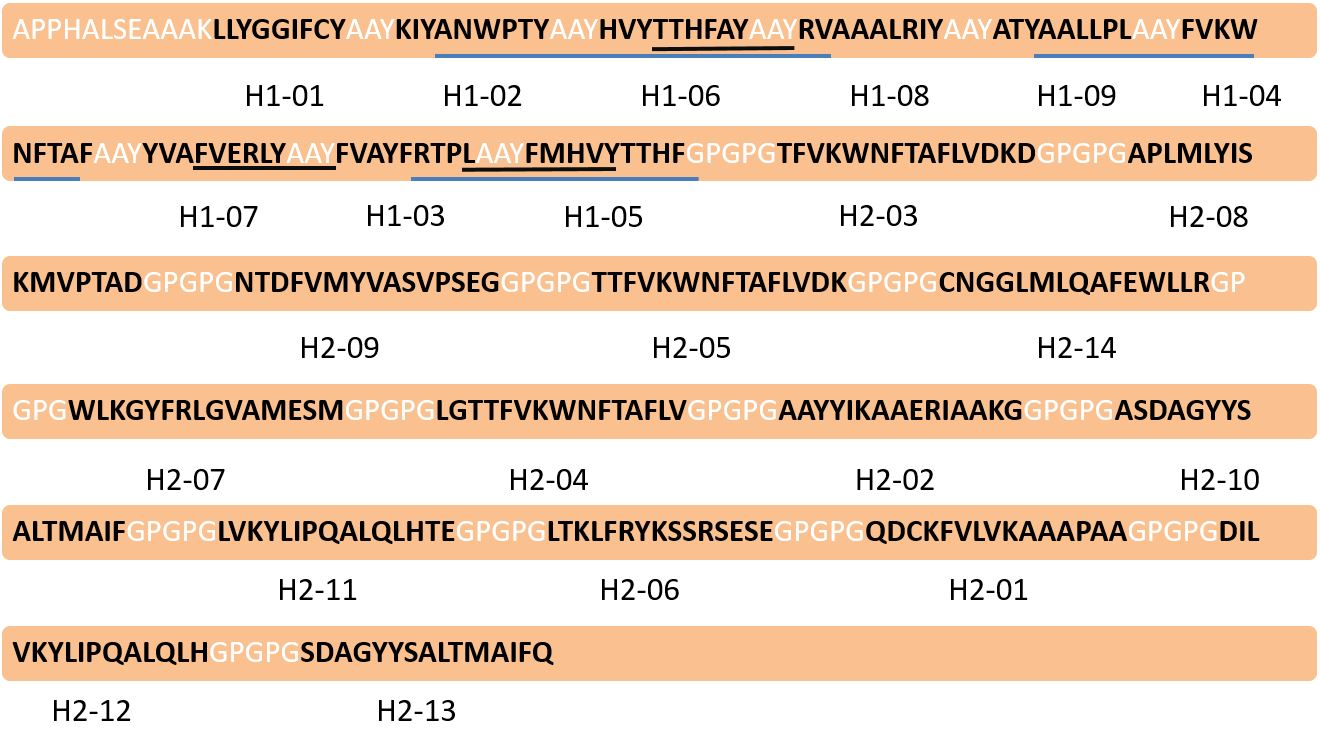

Supplement: Supplementary file 3 — Additional file 3: Figure S1. Proposed vaccine construct. The peptide adjuvant precedes CTL (H1) and HTL (H2) epitopes. Non-specific CTL epitopes and IL-10 inducing HTL epitopes are underlined in black and blue, respectively. [file 13071_2020_4064_MOESM3_ESM.tiff]

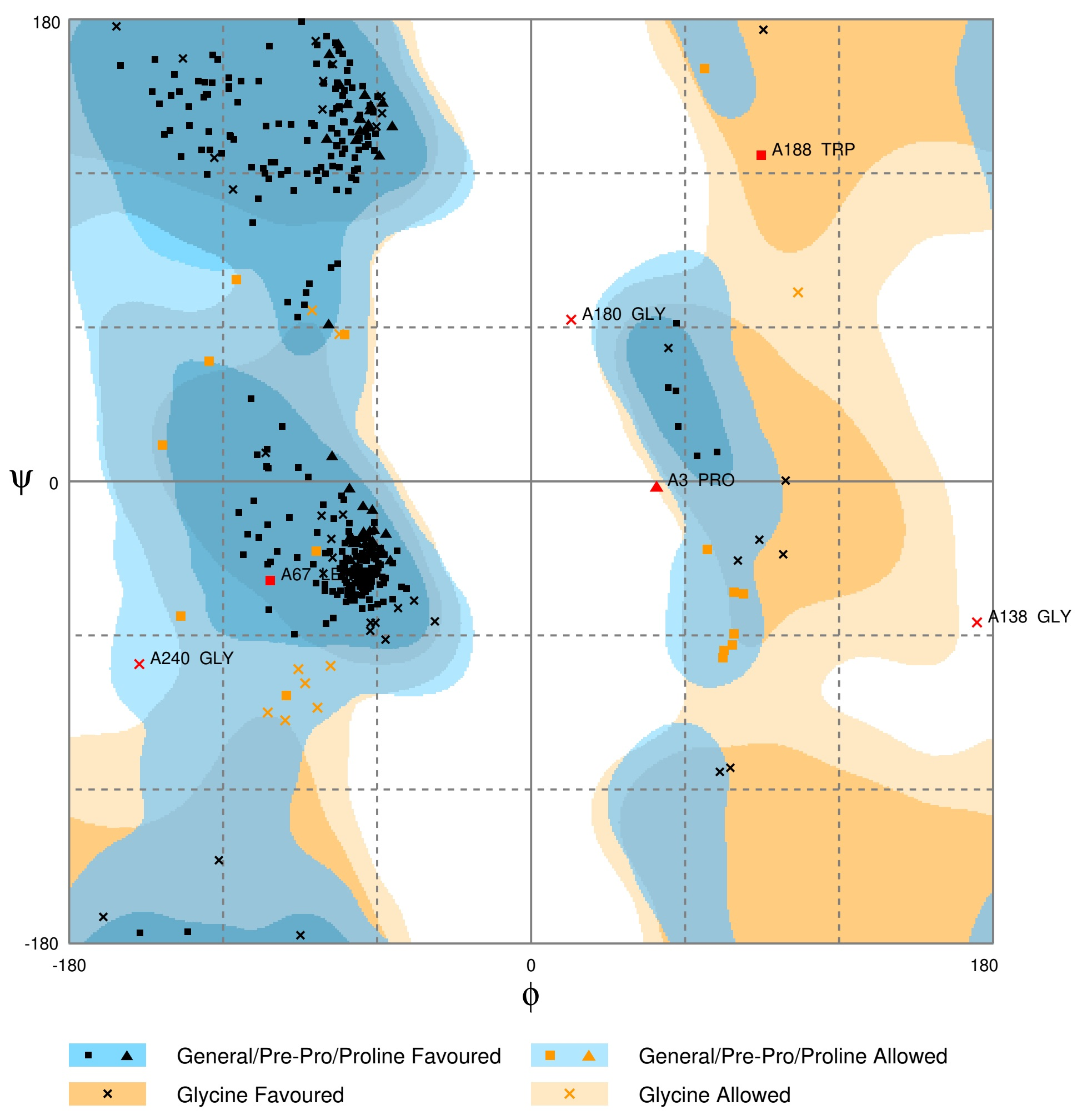

Supplement: Supplementary file 4 — Additional file 4: Figure S2. Ramachandran plot of the refined structure of the vaccine construct. [file 13071_2020_4064_MOESM4_ESM.tiff]

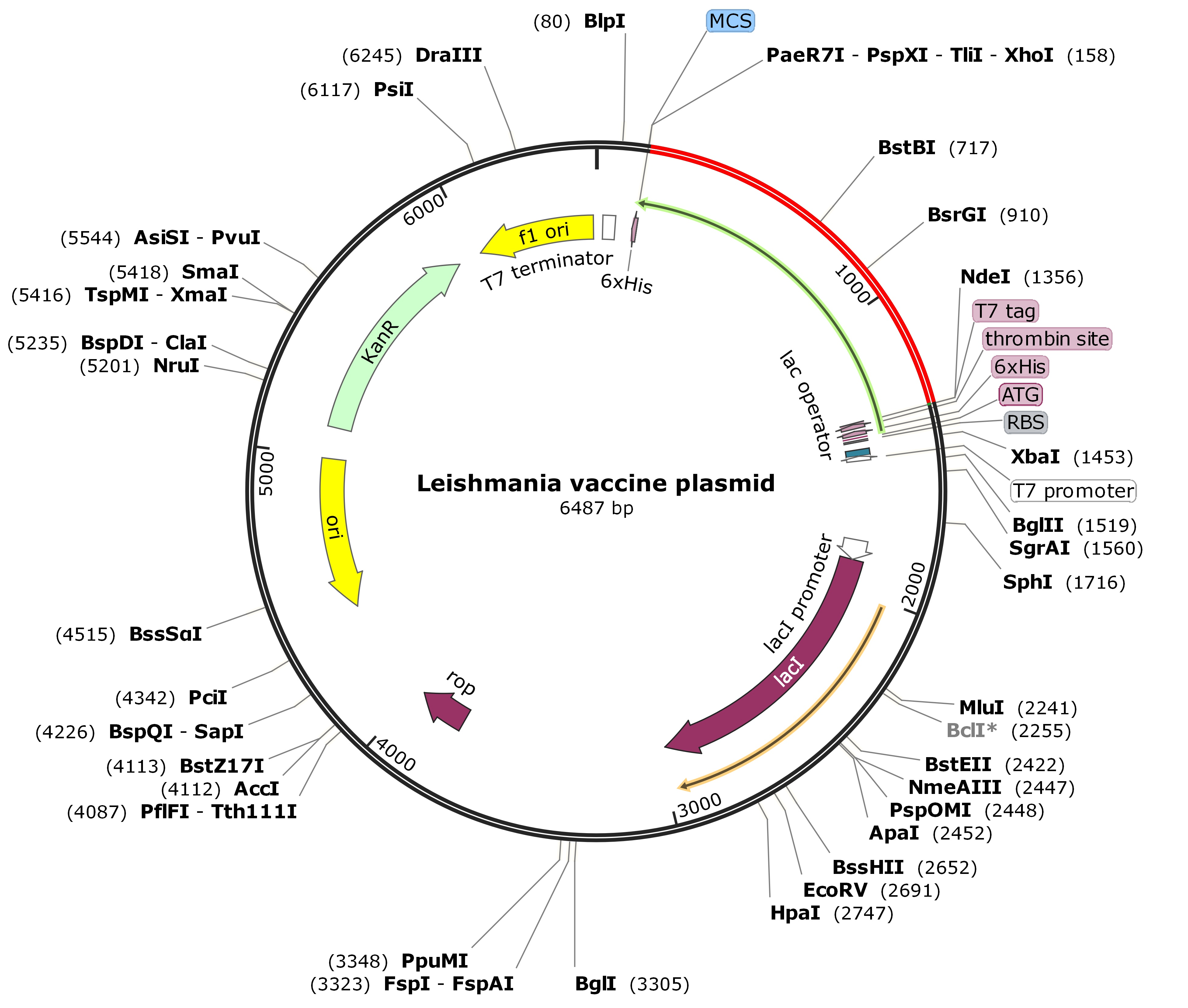

Supplement: Supplementary file 6 — Additional file 6: Figure S3.In silico restriction cloning of vaccine construct. The vaccine coding region is colored red and the green arrow underneath indicates direction of transcription of open reading frame. [file 13071_2020_4064_MOESM6_ESM.tiff]

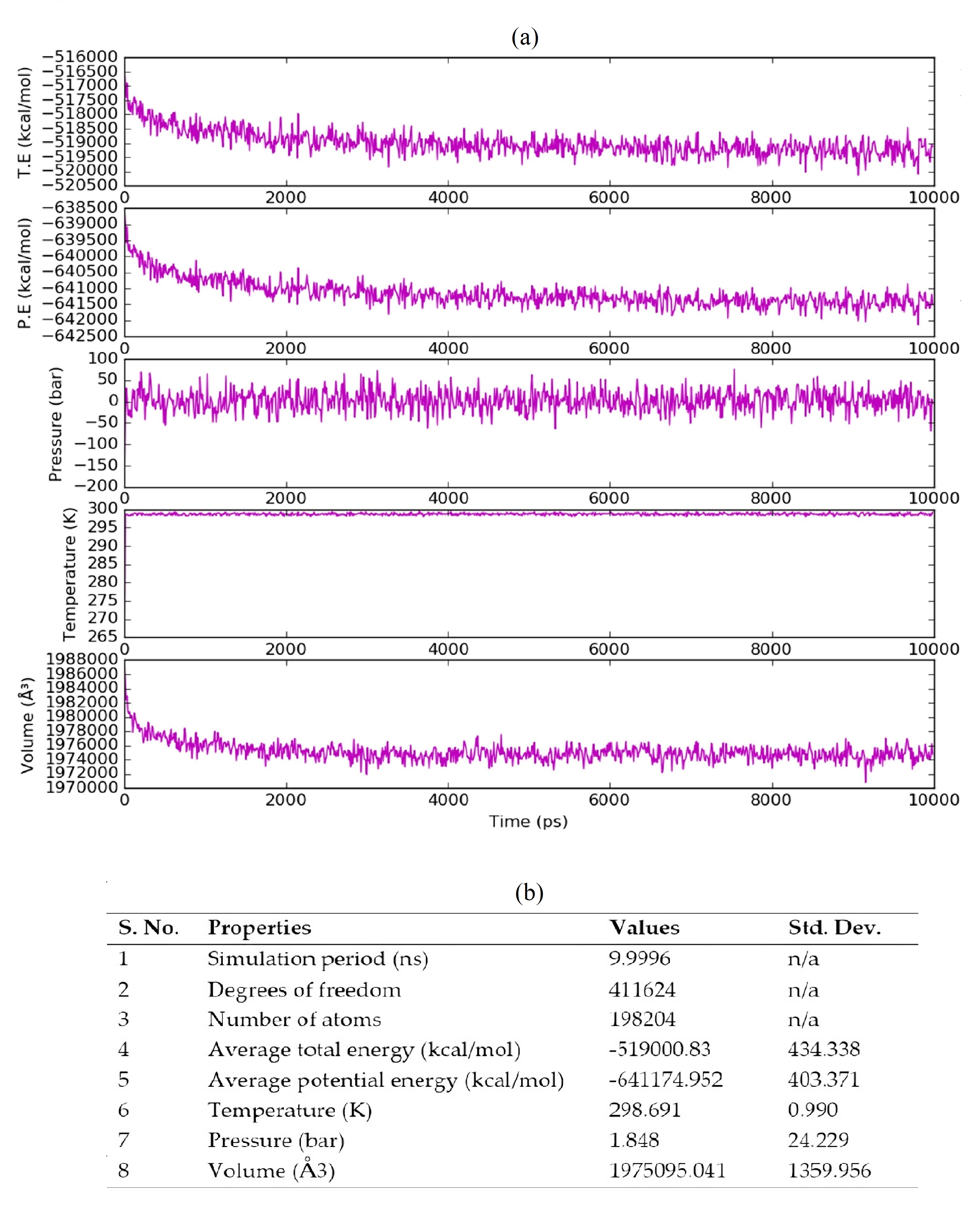

Supplement: Supplementary file 7 — Additional file 7: Figure S4. Simulation quality analysis. a The plot of thermodynamic properties as a function of simulation time over a period of 10 ns. b Tabular summary of thermodynamic properties. Abbreviations: T.E, total energy; P.E, potential energy. [file 13071_2020_4064_MOESM7_ESM.tiff]

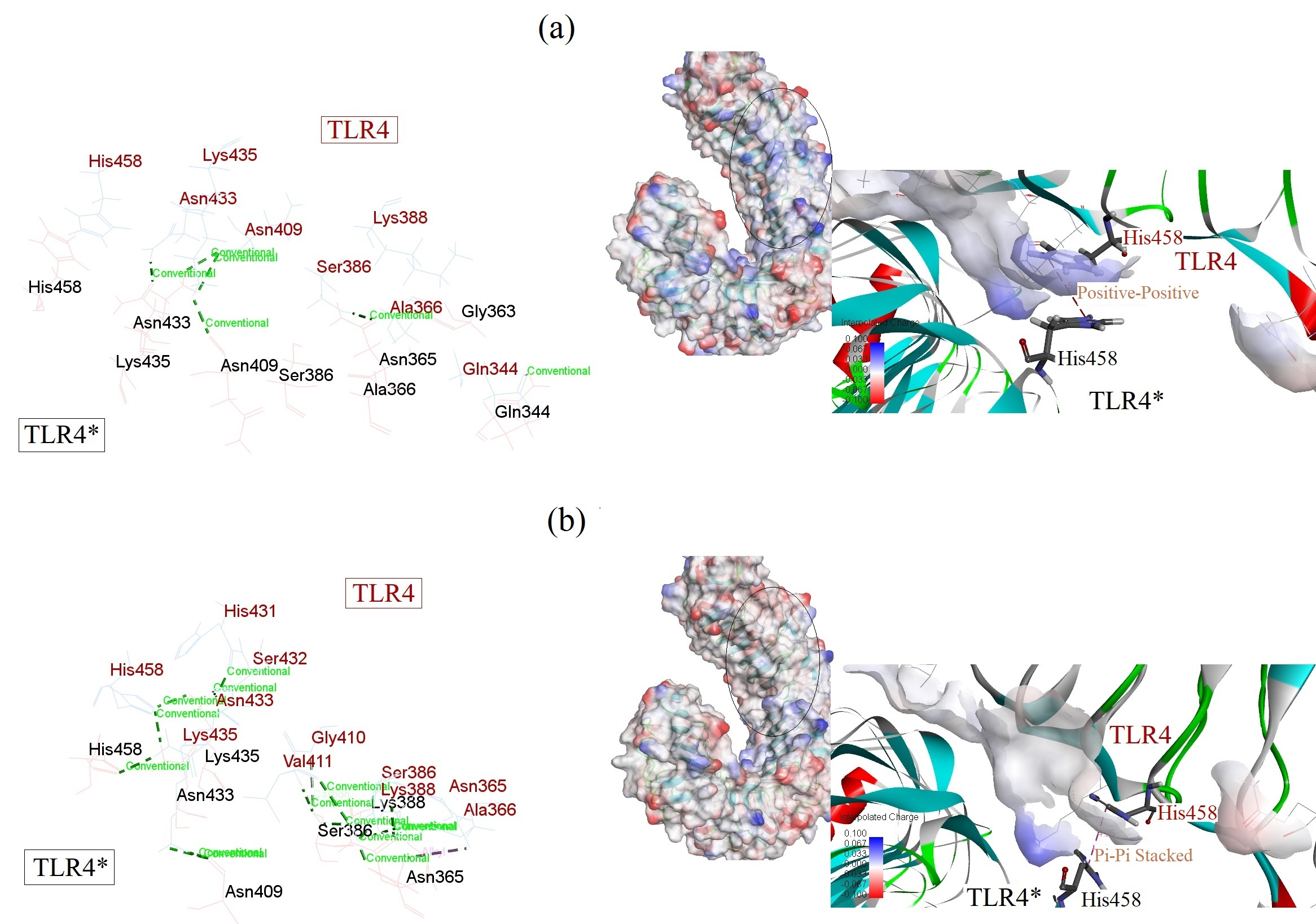

Supplement: Supplementary file 8 — Additional file 8: Figure S5. Bond re-arrangement at the C-terminal domain of TLR4-TLR4* junction. a Vaccine-unbound interface. b Vaccine-bound interface (post-simulation). Hydrogen bonds are represented as conventional bonds. Interacting surface of TLR4 (encircled) highlights residual charges ranging from positive (blue) to negative (magenta). Inset pictures (right) indicate a transition in the mode of interaction between His458 and His458*. [file 13071_2020_4064_MOESM8_ESM.tiff]

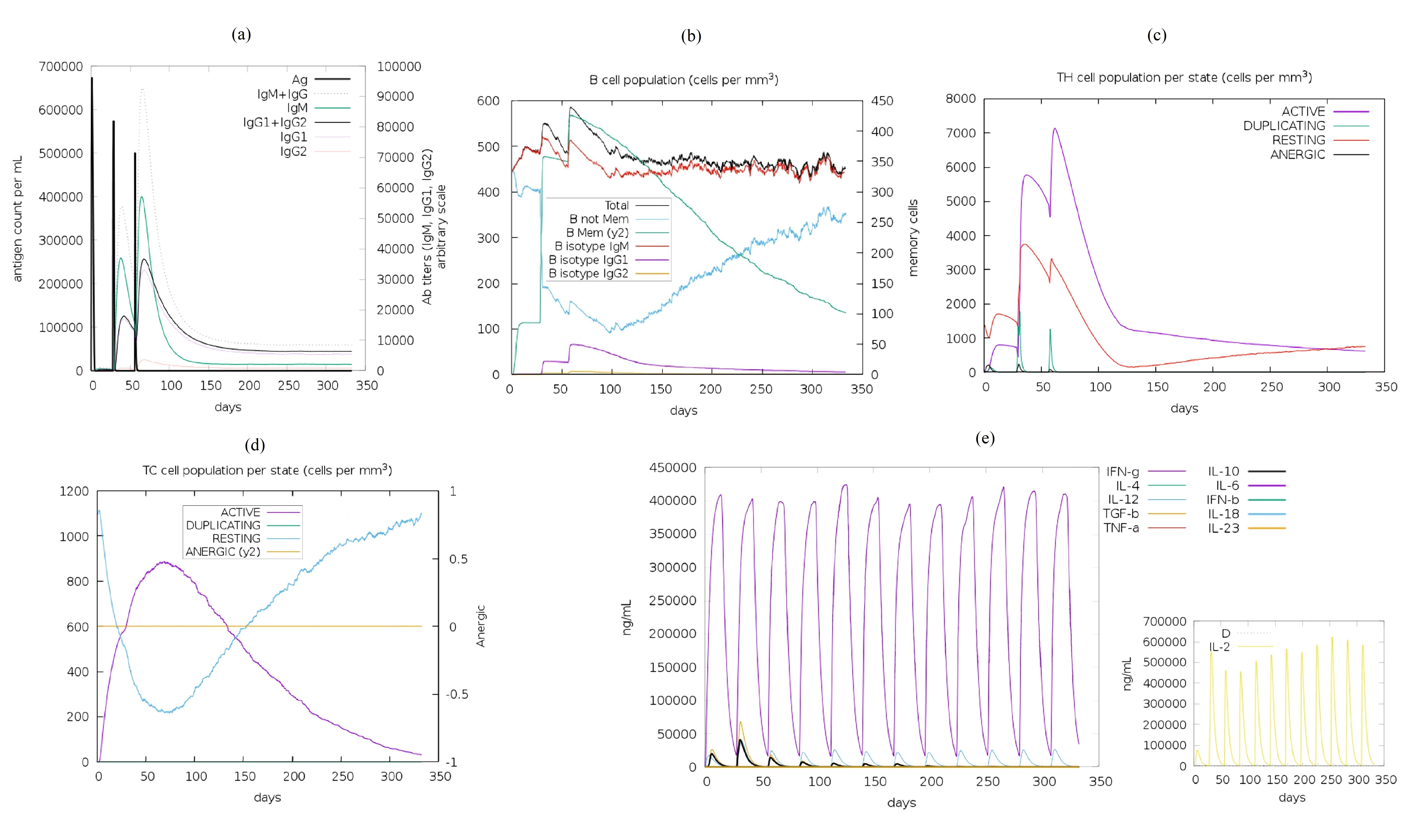

Supplement: Supplementary file 9 — Additional file 9: Figure S6. Simulated immune response following hypothetical immunization. a Immunoglobulin production. b B cell response and memory development. c TH (helper T) cell population per state. d TC (cytotoxic T) cell population per state. e Cytokine levels at regular dose intervals for 12 doses. The inset plot indicates the level of leukocyte growth factor (IL-2) and the potential for clonal expansion (D) after each dose. [file 13071_2020_4064_MOESM9_ESM.tiff]
