# Supplementary material for: An immunoinformatic approach driven by experimental proteomics: in silico design of a subunit candidate vaccine targeting secretory proteins of Leishmania donovani amastigotes
Source: Parasit Vectors. 2020 Apr 15;13:196. doi: 10.1186/s13071-020-04064-8 (PMC7160903; doi:10.1186/s13071-020-04064-8)
Supplement: Supplementary file 5 — Additional file 5: Table S1. Account of conformational epitopes in the vaccine construct. [file 13071_2020_4064_MOESM5_ESM.docx]

Table S1: Account of conformational epitopes in vaccine protein.

| **No.** | **Residues in conformational epitopes (highlighted residues denote overlapping linear epitope region in conformational epitopes)** | **Number of residues** | **Score** |
| --- | --- | --- | --- |
| 1 | A:T123, A:F124, A:V125, A:K126, A:W127, A:N128, A:F129, A:T130, A:A131, A:F132, A:K136, A:D137 | 12 | 0.869 |
| 2 | A:A1, A:P2, A:P3, A:H4, A:S7, A:V153, A:P154, A:T155, A:A156, A:D157, A:G158, A:P159, A:G160, A:P161, A:G162, A:N163, A:T164, A:D165, A:F166, A:V167, A:M168, A:Y169 | 22 | 0.847 |
| 3 | A:Y306, A:L307, A:I308, A:P309, A:Q310, A:A311, A:L312, A:Q313, A:L314, A:H315, A:T316, A:E317, A:G318, A:P319, A:G320, A:P321, A:G322, A:L323, A:T324, A:K325, A:L326, A:F327, A:R328, A:Y329, A:K330, A:S331, A:S332, A:R333, A:P341, A:G342, A:Q343, A:D344, A:C345, A:K346, A:V348 | 35 | 0.844 |
| 4 | A:P68, A:L69, A:A70, A:A71, A:Y72, A:F73, A:V74, A:K75 | 8 | 0.821 |
| 5 | A:G378, A:P379, A:G380, A:P381, A:G382, A:S383, A:A385, A:G386 | 8 | 0.783 |
| 6 | A:Y24, A:V38, A:T40, A:T41, A:H42, A:F43, A:A44, A:Y45, A:A46, A:Y48, A:R49, A:V50, A:A51 | 13 | 0.75 |
| 7 | A:L230, A:G231, A:M234, A:E235, A:S236, A:M237, A:G238, A:P239, A:G240, A:P241, A:G242, A:L243, A:G258 | 13 | 0.729 |
| 8 | A:N251, A:F252, A:T253, A:A254, A:F255, A:L256, A:G260, A:G262, A:A263, A:A264, A:Y265, A:Y266, A:I267, A:K268, A:A269, A:A270, A:E271 | 17 | 0.705 |

.
